# Mathematical model of MMC chemotherapy for non-invasive bladder cancer treatment

**DOI:** 10.3389/fonc.2024.1352065

**Published:** 2024-05-31

**Authors:** Marom Yosef, Svetlana Bunimovich-Mendrazitsky

**Affiliations:** Department of Mathematics, Ariel University, Ariel, Israel

**Keywords:** mathematical oncology, tumor-immune interactions, individual-based model, drug dose determination, non-linear dynamics

## Abstract

Mitomycin-C (MMC) chemotherapy is a well-established anti-cancer treatment for non-muscle-invasive bladder cancer (NMIBC). However, despite comprehensive biological research, the complete mechanism of action and an ideal regimen of MMC have not been elucidated. In this study, we present a theoretical investigation of NMIBC growth and its treatment by continuous administration of MMC chemotherapy. Using temporal ordinary differential equations (ODEs) to describe cell populations and drug molecules, we formulated the first mathematical model of tumor-immune interactions in the treatment of MMC for NMIBC, based on biological sources. Several hypothetical scenarios for NMIBC under the assumption that tumor size correlates with cell count are presented, depicting the evolution of tumors classified as small, medium, and large. These scenarios align qualitatively with clinical observations of lower recurrence rates for tumor size ≤ 30[mm] with MMC treatment, demonstrating that cure appears up to a theoretical *x*[mm] tumor size threshold, given specific parameters within a feasible biological range. The unique use of mole units allows to introduce a new method for theoretical pre-treatment assessments by determining MMC drug doses required for a cure. In this way, our approach provides initial steps toward personalized MMC chemotherapy for NMIBC patients, offering the possibility of new insights and potentially holding the key to unlocking some of its mysteries.

## 1 Introduction

Cancer diseases rank as a leading cause of death and a major health concern in modern society (1). In particular, bladder cancer (BC) is among the most prevalent cancer types in the world, with approximately 573,000 new cases and 213,000 deaths annually (1). The highest incidence rates are observed in Europe, North Africa, West Asia, and North America (2). BC’s high burden on both patients and health-care systems is mainly attributed to its intensive treatment and monitoring requirements, making it one of the most economically costly cancers (3).

Research into the development of cancer cures continues to be a highly challenging process despite tremendous scientific, pharmaceutical, and technological progress in recent decades (4–6). Various studies suggest confronting medical decision-making challenges via collaboration between healthcare professionals monitoring and interpreting data to help diagnose or treat patients, and mathematicians developing new models and computational simulations to characterize tumors and to pave the road to personalized medical treatments (7–12). Taken jointly, these contributions represent a step toward the development of quantitative methods in the complex and nonlinear biology of tumors, in particularly, using differential equations (13, 14).

MMC, epirubicin, and gemcitabine are anti-tumor chemotherapeutic drugs used for non-invasive BC (15, 16). MMC is an anti-tumor antibiotic discovered in the 1950s from *Streptomyces caespitosus* cultures, which selectively inhibits DNA synthesis by cross-linking complementary strands of the double helix, leading to cell death (17). Its large molecular weight limits physiologically systemic uptake in NMIBC, making this drug generally well tolerated (18).

To mathematically analyze BC’s growth under the treatment of MMC chemotherapy, we studied the biological phenomenon of bladder tumor evolution and the current treatment protocols. Over the past decades, the creation of mathematical models for BC treatments has been driven by the need to address shortcomings in existing treatment protocols, such as their limited efficacy and lack of personalization (19–23). Among these, one study conducted by Burgos Simón et al. (19) involved the use of two distinct systems of difference equations. One system models the interactions between tumor cells and inflammatory cells, while the other addresses the subsequent phase involving tumor removal surgery, followed by Bacillus Calmette-Guérin (BCG) immunotherapy treatment. Their model analyzes hospital-sourced data to describe and predict fluctuations in tumor size and immune responses. In Shaikhet and Bunimovich-Mendrazitsky (21), the authors dealt with BC under BCG immunotherapy treatments, and highlighted the importance of the interplay of multiple parameters on the success of immunotherapy. The model in Nave et al. (22) deals with improvement of BCG immunotherapy for BC by adding interleukin 2 (IL-2). By following the complex biological processes of tumor, immune system, and BCG interactions, they provided a reliable platform for *in silico* testing of alternative protocols for BCG instillations and combinations with *IL-2*. While certain aspects of the models mentioned earlier (19–23), such as the law of mass action, form the foundation for our model concerning bladder tumors and immune cells, it’s noteworthy that these models primarily focus on immunotherapy. As a result, our approach to model chemotherapy treatment draws inspiration from the works of de Pillis et al. (24) and Rodrigues et al. (25) who have specifically constructed models addressing chemotherapy treatments.

The work of de Pillis et al. (24) models cancer growth on a cell population level to investigate tumor dynamics. By merging clinical data from both laboratory mice and human trials together with established mathematical terms of cell–cell interactions and Michaelis–Menten, they started from the observation of biologists that cancer growth is controlled by a healthy immune system. Accordingly, they developed an ODEs system (Equation (0)) that follows the stimulation of the immune response by tumor cells under a combination of immune, vaccine, and chemotherapy treatments:


(0) $$\left\{ \begin{array}{l} \frac{\text{d}T}{\text{d}t}=aT(1-bT)-cNT-DT-K_{T}(1-{\text{e}}^{-M})T,\\ \frac{\text{d}N}{\text{d}t}=eC-fN+g\frac{T^{2}}{h+T^{2}}N-pNT-K_{N}(1-{\text{e}}^{-M})N,\\ \frac{\text{d}L}{\text{d}t}=-mL+j\frac{D^{2}T^{2}}{k+D^{2}T^{2}}L-qLT+(r_{1}N+r_{2}C)T\\ -uNL^{2}-K_{L}(1-e^{-M})L+\frac{p_{I}LI}{g_{I}+I}+v_{L}(t),\\ \frac{\text{d}C}{\text{d}t}=\alpha -\beta C-K_{C}(1-e^{-M})C,\\ \frac{\text{d}M}{\text{d}t}=-\gamma M+v_{M}(t),\\ \frac{\text{d}I}{\text{d}t}=-\mu_{I}I+v_{I}(t),\\ D=d\frac{{\left(L/T\right)}^{l}}{s+{\left(L/T\right)}^{l}}. \end{array}\right.$$

At time *t*, the populations are represented by *T*(*t*) for tumor cell population, *N*(*t*) for total NK cell population, *L*(*t*) for total CD8^+^T cell population, *C*(*t*) for number of circulating lymphocytes, *M*(*t*) for chemotherapy drug concentration in the bloodstream, and *I*(*t*) for immunotherapy drug concentration in the bloodstream. The characterization of the biological phenomenon was captured through stability analysis and simulations, revealing cases where disease progression is very sensitive to the initial tumor size or levels of specific immune cells. This model provides new insights into tumor dynamics, especially regarding the desirable tumor-free state.

Rodrigues et al. (25) proposed an ODEs-based model to study the chronic lymphocytic leukemia (CLL)-immune dynamics under chemoimmunotherapy. Similar to the approach of de Pillis et al. (24), they highlighted the role of the immune system in eliminating cancer cells. This model provides functional structures for chemotherapy terms with saturating behavior, allowing to model constant and periodic drug instillation, that can be applied to every drug that is given periodically. A critical factor in the success of chemotherapy, as shown in their model and observed by clinicians (26, 27), is the intensity with which the tumor cells stimulate immune cell production. This study serves as the foundation for the formulation of chemotherapy terms in our model.

Realistic and physical modeling of the bladder has been studied thoroughly in the context of hyperthermia of MMC treatment for NMIBC, which is a targeted heating of the tumor area (28–31). For example, the authors in Schooneveldt et al. (28) utilized a convective thermophysical fluid model, based on the Boussinesq approximation to the Navier–Stokes equations, to assess the benefits of physically accurate fluid modeling in NMIBC patients undergoing hyperthermia treatment. Their analysis, based on Computed Tomography (CT) scans from 14 BC patients, demonstrated a significant improvement in temperature prediction accuracy within the urinary bladder compared to previous model (29). Additionally, Sadée and Kashdan (31) developed a mathematical model using conductive Maxwell’s equations to simulate therapy administration and the Convection-Diffusion equation for incompressible fluid to study heat propagation through bladder tissue. However, despite the progress made by biological and mathematical studies of MMC chemotherapy for BC treatment, the optimal dose and mechanism of this drug are yet to be determined (18, 32). To the best of our knowledge, the fundamental oncological aspects of tumor-immune dynamics for this purpose are absent in the existing studies.

In the current work, we recapitulate the state of the art of mathematical modeling by presenting in this research a novel mathematical model describing the dynamics of BC considering MMC chemotherapy. The proposed model is based on the solution of a system of three nonlinear ODEs that describes the tumor-immune dynamics under the assumption that MMC chemotherapy is administered continuously at a low dose. Notably, in a biologically feasible parameter regime, a stable tumor-free equilibrium with a non-trivial structure exists. Moreover, we utilized this stability condition of this equilibrium for the creation of a new method of personalized drug dosage determination. There are obvious practical implications for chemotherapy treatment associated with the ability to calculate MMC dose. To evaluate our work, we compared the model simulations with clinical data. In addition, a sensitivity analysis was conducted to observe how variations in the estimated parameters affect the count of tumor cells. Ultimately, we discuss some more general aspects of the therapeutic process.

The rest of the paper is organized as follows: Section 2 introduces the underlying biological processes of BC dynamics under MMC chemotherapy treatment, that the model aims to describe. Section 3 is devoted for the mathematical model formulation. Section 4 presents the model analysis, including the model’s steady states and their clinical impact. The results are discussed in Section 5- where necessary conditions for homeostasis and tumor elimination are presented, and a new method is proposed for determining a theoretically patient-specific upper bound of MMC dose. Finally, Section 6 concludes the biological and mathematical features of the model, including its clinical potential.

## 2 Biological background

NMIBC is defined as a growth of malignant tumor cells in the urinary bladder, superficially developing on the inner surface and confined to the mucosa or submucosa layers of the bladder wall (33). The major risk factors are tobacco smoking and specific chemical exposures in the occupational and general environments (34).

The treatment of BC comprises two phases, transurethral resection (TUR) and adjuvant therapy. TUR is the standard initial treatment involving an endoscopic procedure to remove the visible tumor (35). Although TUR alone is capable of eradicating tumors completely in some cases, these tumors tend to recur and may progress to muscle-invasive BC (MIBC) (16, 36). In an attempt to extend the recurrence-free interval, TUR is followed by the administration of adjuvant therapy, in the form of intravesical treatment with immunotherapy or chemotherapy, which is given via the catheter (16, 36). The focus of our model is on the chemotherapeutic drug MMC. MMC is instilled in the bladder via the catheter and is generally safe due to its limited effects on the bladder (18, 37). After instillation, MMC acts by inhibiting DNA synthesis and subsequent death of tumor cells (38).

Despite improved management for decreasing the recurrence rate and prolonging the progression-free interval, the clinical effectiveness of MMC is often limited, as the estimated 5-year recurrence rate varied between 49% to 75%, with a risk of progression to the muscle-invasive stage (38–43). Moreover, for this treatment modality, the length and frequency of repeat chemotherapy instillations are still controversial (38–43). Clinicians emphasize that the inability to define drug dosage, treatment frequency, and duration plays a significant role in the failure of MMC treatment, highlighting the absence of an ideal MMC regimen as a key issue (18, 44). One way that has shown enhanced efficacy of MMC is treatment via microwave-induced hyperthermia of MMC, but the success of this treatment is also limited (45, 46). Therefore, new strategies are needed to improve treatment protocols. As the initial step in working towards a new strategy, we establish the first mathematical model to encompass both immunological aspects and MMC chemotherapy for BC treatment, conducting analytical investigations incorporating various model assumptions. In this preliminary phase, we will describe BC elimination under MMC chemotherapy.

**Figure 1:**
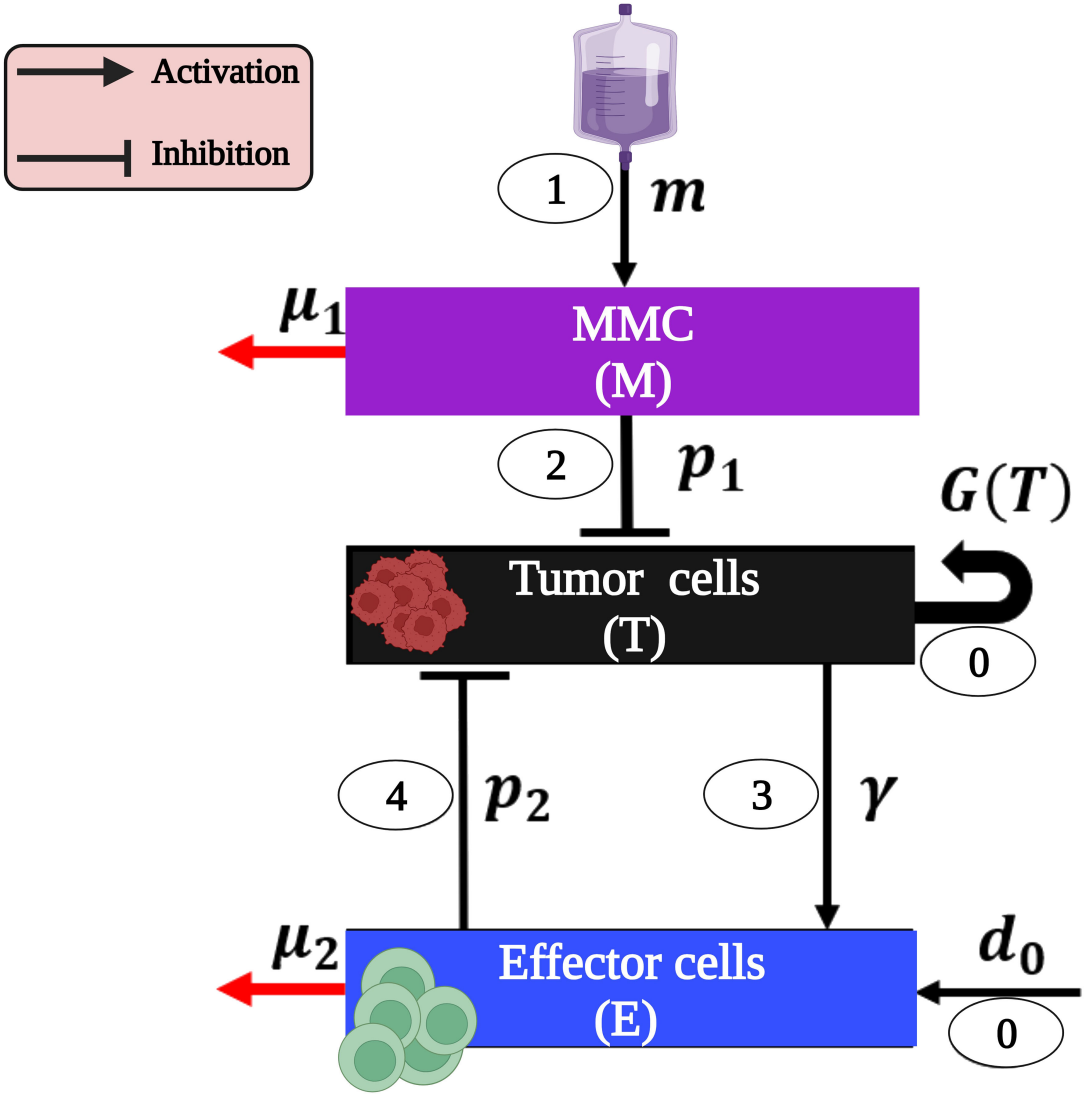
The model interactions of BC cells and immune system as a result of MMC chemotherapy (presented with parameters and the cascade steps are numbered inside ovals as outlined in the text). Note that parameters without a direct influence from one cell type to another (*p*_3_) or those defined solely by detailed mathematical terms in system (1) (*r,k,a*) are exclusively present in system (1). This image was created with BioRender.com.

The cascade of events leading to BC eradication after MMC treatment can be summarized as follows (see **Figure 1**): BC tumor cells, termed here as (T) undergo proliferation (47–51), and effector cells (*E*), are produced at a constant rate due to healthy body homeostasis, i.e., bladder functions are kept within a normal range (step 0, **Figure 1**) (52, 53).

MMC (*M*) instillation (step 1, **Figure 1**) results in a cascade effects of tumor elimination:

1. **The direct effect:** The MMC-DNA interaction leads to the inhibition of DNA synthesis and the subsequent influences on the process of cell division. Consequently, a fraction of urothelial tumor cells (T) undergo arrest of the cell cycle and apoptosis (step 2, **Figure 1**) (54–57).
2. **The indirect effect:** effector cells (E) are activated by apoptotic tumor cells (T) (step 3, **Figure 1**) and eliminate the latter via DCs’ phagocytosis and Cytotoxic T cells’ (CTLs) cytotoxicity (step 4, **Figure 1**) (55, 58). This suppression of tumor cells (T) leads to the destruction of the entire tumor (54, 55).

## 3 Materials and methods

### 3.1 Formulation of model equations

Our mathematical model consists of a system of ODEs that describes interactions between tumor cells and the immune system under MMC chemotherapy. Specifically, we track the temporal dynamics of the following three populations: MMC chemotherapy drug dose, *M*(*t*); bladder tumor cells population, *T*(*t*); and effector cells population, *E*(*t*). The formulation of the equations was done by bringing together the specific forms of cell growth, cell-cell interactions, and drug-cell interactions (see **Figure 1**):

**Equation for the chemotherapeutic agent MMC (M).** MMC (*M*) is delivered into the bladder cavity for 1-2 hours by once-a-week instillations over a 6-week to 8-week period and then once monthly for 1-year (32, 59–61). Processes of this type introduce time-dependent discontinuities into the model. The resultant non-autonomous system may present analytical complexity. Therefore, at the present stage, we have chosen to simplify and model constant administration of MMC to the bladder; *m* ≥ 0 is a constant parameter of the MMC instillation rate. We obtain Equation (1a):


(1a) $$\frac{dM}{dt}=-\underset{\text{washout}}{\underset{︸}{\mu_{1}M}}+\underset{\text{source }}{\underset{︸}{m}},$$

where *µ*_1_ is the washout rate of MMC (*M*).

**Equation for the tumor cells (T)**. Bladder tumor cells (*T*), known for their heterogeneity (62), are assumed here to be entirely identical for simplification. These cells undergo apoptosis due to direct cytotoxicity of MMC (*M*) by inhibiting DNA synthesis (38, 54, 56, 57). Hence, tumor cells,(*T*), must decrease at an intensity that is proportional to their encounter with MMC (*M*). By assuming these dynamics have a Michaelis-Menten form with *p*_1_ as a rate constant, and a half-saturation parameter *a*, we get the term $\frac{p_{1}TM}{M+a}$ . Upon activation by tumor cells’ (*T*) apoptosis, effector cells (*E*) begin with the subsequent engulfment and destruction of the tumor cells (*T*) (54, 55, 58), at a constant rate *p*_2_. Based on these processes, the tumor dynamics can be formulated as follows in Equation (1b):


(1b) $$\frac{dT}{dt}=-\underset{\text{killed by MMC }}{\underset{︸}{\frac{p_{1}MT}{M+a}}}-\underset{\text{killed by immune cells }}{\underset{︸}{p_{2}ET}}+\underset{\text{growth }}{\underset{︸}{rT\left(1-\frac{T}{k}\right)}},$$

Here, the number of tumor cells (*T*) increases due to natural growth, as indicated by the third term following the logistic growth law; *r* is the tumor growth rate and *k* represents the tumor’s carrying capacity. In this context, we suggest that the carrying capacity *k* represents a state of no-cure or even death within the biological context. Hence, our focus is only on the range 0 ≤ *T* ≤ *k*.

**Equation for the effector cells (E)**. CTLs and dendritic cells (DCs) are immune cells represented by the term effector cells (*E*). DCs reside in the bladder (63), so we assume that effector cells (*E*) proliferate at a constant rate *d*_0_, in alignment with previous mathematical models that describe antigen-presenting cells (APCs) (64, 65). Apoptotic urothelial tumor cells (*T*), given by the term $\frac{p_{1}TM}{M+a}$ , induce the activation of effector cells (*E*) at a constant rate *γ* (54, 55, 58). The Michaelis-Menten form of this term accounts for the limited nature of this recruitment (66). In addition, the term *p*_3_ *TE* describes the effector cells' (*E*) deactivation via an encounter with tumor cells (*T*) (54, 55). The natural mortality rate of effector cells is *µ*_2_. Hence, effector cells (*E*) satisfy the following Equation (1c):


(1c) $$\frac{dE}{dt}=\underset{\text{source }}{\underset{︸}{d_{0}}}-\underset{\text{deactivation }}{\underset{︸}{p_{3}ET}}-\underset{\text{death }}{\underset{︸}{\mu_{2}E}}+\underset{\text{activation }}{\underset{︸}{\gamma \frac{p_{1}TM}{M+a}}}.$$

Thus, the tumor-immune interactions of NMIBC under MMC chemotherapy are modeled by the following system of ODEs:


(1) $$\{\begin{array}{l} \frac{dM}{dt}=-\mu_{1}M+m,\\ \frac{dT}{dt}=-T\left(\frac{p_{1}M}{M+a}+p_{2}E\right)+G\left(T\right),\\ \frac{dE}{dt}=d_{0}+\gamma \frac{p_{1}TM}{M+a}-E\left(p_{3}T+\mu_{2}\right). \end{array}$$

Where:


$$G\left(T\right)=rT\left(1-\frac{T}{k}\right)$$

With initial conditions:


M(0) ≥ 0,        T(0) ≥ 0,         E(0) ≥ 0.

Note that the requirement above that for all *t* ∈ [0,∞),0 ≤ *T*(*t*) ≤ *k*, is just a biological constraint of the model. Without assigning a specific value to bound the tumor cell count, *k* and, consequently, *T*(*t*) can theoretically represent any number of tumor cells.

### 3.2 Estimation of parameters

For the model to be complete, we carefully estimated parameter ranges so that they are realistic and correspond to values found in biological studies, while some were determined by the model to yield meaningful results in simulations, consistent with reported phenomena in the literature (see **Table 1**). The parameters, drawn from animal models, cell lines, and human tumor samples, are utilized for exploratory model analysis. Therefore, it is important to note that it is unsafe to use them in clinical settings with human cancer patients, as refinement through separate work based on clinical research findings is mandatory before further utilization. The interpretation of all parameters below should be considered within the context of their respective roles in the ODEs system (Equation 1).

**Table 1:** List of the model variables and parameters descriptions.

Variable	Description	Unit	
**M(t)**	MMC chemotherapy drug amount at time *t*	[*μ*M]	
**T(t)**	Bladder tumor cells population at time *t*	Cells
**E(t)**	Effector cells population at time *t*	Cells
Parameter	Description	Estimate	Source
*µ*_1_	Decay rate of MMC	21.05[*day* ^−1^]	(67)
*m*	Instillation rate of MMC	6.561 [*µM/day*]	(32, 59), Calculated
*r*	proliferation rate of tumor cells (*T*)	(0.01-0.045)[*day* ^-1^]	(20)
*k*	Carrying capacity of tumor cells (*T*)	(0.09-1)×10^9^[*cells*]	(20, 68)
*p*_1_	Inhibition rate of (*T*) by MMC	(0.12-0.2)[*day* ^-1^]	(56)
*a*	Half-saturation constant	1×10^2^[*μM*]	Estimated
*p*_2_	(*T*) cells inhibition rate by (*E*) cells	(3.7-5.5)×10^-6^[*cells* ^-1^ *day* ^-1^]	(22, 69)
*d*_0_	Constant production rate of effector cells	1.032×10^5^[*cells*×*day* ^-1^]	(52)
γ	Effector cells' (*E*) activation rate	9.12[*day* ^-1^]	(70)
*μ*_2_	Effector cells' (*E*) death rate	9.12[*day* ^-1^]	(24)
*p*_3_	Effector cells' (*E*) deactivation rate	(1.1-1.59)×10^-6^[*cells* ^-1^ *day* ^-1^]	(24)

All parameters used are strictly positive.

• **The decay rate of MMC - *µ*_1_:**

Removal by metabolism, tissue binding and minimal absorption across the bladder epithelium are the primary factors for MMC (*M*) decay in urine (16, 67, 71). MMC (*M*) elimination rate *µ*_1_ = 21.05 [*day^-1^*] is derived from the mean constant rate of absorption and degradation, reported by Dalton et al. (67). From the assumption of exponential decay, the biological half-life of MMC (*M*) is $t_{1/2}=\frac{\ln2}{21.05[day^{-1}]}=47.4$ minutes. There have been numerous studies that support this result (37, 72, 73). One of them, the work of Gao et al. (37), confirms that approximately 6% − 16% of the MMC dose was present in the bladder tissue at the end of the 2 hours treatment period [see **Table 1** in (37)]. Even when using the same sources, MMC half-life can vary, because there is a substantial intra and inter-patient variability in degradation and absorption constant rates based on various factors, such as incomplete bladder emptying during treatment, urine acidity and hydration status (72, 73).

• **The MMC instillation rate - *m:***

A widely accepted protocol for a single MMC treatment session is 40 mg in 50 ml sterile water administered intravesically for 2 hours (74–77). Therefore, we considered the corresponding drug dose, *m*_0_, that was calculated as follows (the molecular weight of MMC is 334 g/mol (78)): *m*_0_ = (Protocol dose)×(Mw of MMC) $=\left(\frac{40\text{ mg}}{50\text{ ml}}\right)\;\left(\frac{\text{g}}{10^{3}\text{ mg}}\right)\;\left(\frac{1\text{mol}}{334\text{ g}}\right)\;\left(\frac{10^{6}\mu \text{mol}}{1\text{ mol}}\right)=2.395[\mu mol/ml]=2,395[\mu M]$ . Given the hypothetical nature of continuous drug administration for hundreds of days, we used a relatively small amount of drug to preserve a total amount similar to that given in a single treatment. This strategy aims to simplify the real two-hour treatment, which is generally considered safe for the local administration area (18, 37), and may reduce the risk of theoretical over-toxicity. Over-toxicity is indirectly addressed in this work through the consideration of drug doses below the highest recommended values in the literature, as discussed in the results section. Therefore, we chose to scale by *τ* = 365 days which is close to the number of simulated days, to get the drug instillation rate: $m=\frac{m_{0}}{\tau }=\frac{2,395[\mu M]}{365\;days}=6.561[\mu M/day]$ . We chose to use these units because they are compatible with *in vitro* experiments that were done by Ojha et al. (56, 79). While practical pulsed therapy will be discussed in future work, the rationale for assuming and supporting the validity of our approach in scaling the drug instillation rate, including simulations comparing the effects of this administration on large and low MMC doses, is provided in Subsection 4.2 of the **Supplementary Information**.

• **The inhibition rate of urothelial tumor cells by MMC- *p*_1_**:

According to Ojha et al. (56), a 24 hours exposure to 5*µ*M of MMC (*M*) induced 12-20% apoptotic cell death in non-invasive tumor tissue samples from patients. Hence, a reasonable first estimate is the range:


$$p_{1}=(0.12-0.20)\;[day^{-1}]$$.

• **The saturation of the killing effect on urothelial tumor cells *by MMC* - *a***:

The half saturation constant was calculated from model simulations, to be 100[*µM*]. This theoretical value facilitates scenarios representing tumor persistence and elimination with treatment, and the observed activation of effector cells' anti-tumor activity through modulation of anti-tumor immunity compared to the no-treatment case (55, 58).

• **The constant production rate of *Effector cells* - *d_0_***:

We use the information that during homeostasis, DCs are the only subset of effector cells (*E*) which is capable of continuous replacement with new cells (53). DCs undergo a limited number of divisions in the spleen or lymph nodes, and are replenished at a rate of nearly 4.3 × 10^3^ cells per hour (52). Hence, $d_{0}=4.3\times 10^{3}[cells\times hour^{-1}]=1.032\times 10^{5}[cells\times day^{-1}]$ . The explanation for assuming constant effector cell production and the rationale behind choosing this value are provided in Subsection 4.1 of the **Supplementary Information**.

## 4 Model analysis

We establish the biological validity of the model through positive invariance property, i.e., every solution of the system (Equation (1)) with positive initial conditions remains in the positive orthant ${\mathbb{R}}_{+}^{3}$ . The invariance of positive orthant is quite useful for us to formally verify the safety properties of our dynamical system. Since the model variables describe biological and chemical elements represented by nonnegative values in real processes, it is important that we do not obtain negative values. Thus, we first prove the positivity of the solutions (see proof in the **Supplementary Information**). Next, the use of positive invariance property allows us to explore the model’s equilibrium points from a biological perspective, including their stability analysis and an oncological interpretation to formulate the desired mathematical conditions for cure.

### 4.1 Steady states and stability analysis

The model [system (1)] is characterized by four nonnegative equilibria (see **Supplementary Information** for details on the steady-state derivation). In the absence of a straightforward biological interpretation for the parametric form of the cancer equilibria, we decided to analyze their stability numerically in the **Supplementary Information**, and to focus solely on disease-free equilibrium points which are summarized in **Table 2**. The stability analysis is performed for the model with and without chemotherapy. For convenience, *I*_1_ and *I*_2_ denote the terms $\frac{p_{2}d_{0}}{\mu_{2}},\frac{p_{1}\frac{m}{\mu_{1}}}{\left(\frac{m}{\mu_{1}}+a\right)}$ , respectively, when appeared in the text.

**Table 2:** Summary of the stability characteristics for the nonnegative equilibria solutions of system (1), in the absence of chemotherapy (*m* = 0) and with therapy (*m >* 0).

Treatment	Equilibriua	M^∗^	T^∗^	E^∗^	Stability
*m* = 0	*EB*_1_	0	0	$\frac{d_{0}}{\mu_{2}}$	*r < I*_1_ locally stable
*m >* 0	*EB*_2_	$\frac{m}{\mu_{1}}$	0	$\frac{d_{0}}{\mu_{2}}$	*r < I*_1_ + *I*_2_ locally stable

No point is shared by both cases.

With the formulation described in the **Supplementary Information**, we can now investigate the local stability of the linearized model by studying the Jacobian matrix *J* of the system, given by Equation (2):


(2) $$J=\left[\begin{array}{ccc} \frac{\partial F_{1}}{\partial M} & \frac{\partial F_{1}}{\partial T} & \frac{\partial F_{1}}{\partial E}\\ \frac{\partial F_{2}}{\partial M} & \frac{\partial F_{2}}{\partial T} & \frac{\partial F_{2}}{\partial E}\\ \frac{\partial F_{3}}{\partial M} & \frac{\partial F_{3}}{\partial T} & \frac{\partial F_{3}}{\partial E} \end{array}\right]=\left[\begin{array}{ccc} -\mu_{1} & 0 & 0\\ -\frac{p_{1}T}{M+a}+\frac{p_{1}TM}{{\left(M+a\right)}^{2}} & r-\frac{2r}{k}T-p_{2}E-\frac{p_{1}M}{M+a} & -p_{2}T\\ \frac{\gamma p_{1}T}{M+a}-\frac{\gamma p_{1}TM}{{\left(M+a\right)}^{2}} & -p_{3}E+\frac{\gamma p_{1}M}{M+a} & -p_{3\;}T-\mu_{2} \end{array}\right].$$

The following subsections present a stability analysis of the nonnegative equilibria: first, in the absence of chemotherapy (4.1.1), and second, under continuous therapy (4.1.2).

#### 4.1.1 Homeostasis equilibrium (without treatment)


$$EB_{1}=\left(M^{*},T^{*},E^{*}\right)=\left(0,\;0,\;\frac{d_{0}}{\mu_{2}}\right),$$

At this equilibrium, there are no tumor cells, and the immune cells exhibit a homeostatic net production value dictated by the ratio between constant production and natural mortality of these cells, *E* ^∗^. This value, regardless of stability condition to be discussed separately, reflects the equilibrium itself as a non-adverse event, as the killing rate of tumor by immune cells, *p*_2_, is not a factor in *EB*_1_. Therefore, we conclude that the bladder maintains homeostasis.

The eigenvalues of the Jacobian evaluated at equilibrium are: $\overset{¯}{\lambda }=[-\mu_{1};-\frac{d_{0}p_{2}}{\mu_{2}}+r;-\mu_{2}]$ .

Thus, all eigenvalues are negative if:


(3) $$-\frac{d_{0}p_{2}}{\mu_{2}}+r<0,\Rightarrow r<\;\frac{d_{0}p_{2}}{\mu_{2}}.$$

Hence, if the condition in Equation (3) is satisfied, then every solution of the original system (1) that starts near *EB*_1_ converges to *EB*_1_ as *t* → ∞. That is, the homeostasis equilibrium *EB*_1_ equilibrium is locally asymptotically stable when *r < I*_1_ and unstable when *r > I*_1_. Simulations in **Supplementary Figure S1** illustrate that the tumor rapidly disappears for parameters that satisfy the stability criterion.

From an oncological perspective, the term *I*_1_ can be interpreted as the intensity of tumor killing by the immune system [more specifically, effector cells (*E*)]:

1. The numerator, *d*_0_ *p*_2_, is the product of the daily production rate of effector cells (*E*), and the daily killing rate of tumor cells (*T*) by effector cells (*E*), respectively. Therefore, this term reflects the potential daily killing rate of tumor cells (*T*).
2. The net daily rate of this killing process is obtained as *d*_0_ *p*_2_ is divided by the mortality rate of effector cells (*E*), *µ*_2_.

Accordingly, stability condition (3) characterizes the range of the tumor growth rate *r*, for which immune activity *I*_1_ is capable of killing tumor cells, to a stage where the immune system is in homeostasis. For smaller values of *I*_1_, the tumor’s growth rate dominates and the tumor-free equilibrium destabilizes.

#### 4.1.2 Tumor-free equilibrium (under treatment)


$$EB_{2}=\left(M^{*},T^{*},E^{*}\right)=\left(\frac{m}{\mu_{1}},\;0,\;\frac{d_{0}}{\mu_{2}}\right).$$

At this point, no tumor cells are present, and the immune system is in homeostasis, as outlined for *EB*_1_. One might wonder, however, how an equilibrium in which chemotherapy is present in the system can indicate a cure for a disease. We resolve this issue by referring back to the model’s structure. The term $\frac{m}{\mu_{1}}$ reflects a very small amount of MMC. Recall that the instillation rate, *m*, is obtained by division of the drug dose *m*_0_, by *τ*, which is close to the number of simulated days. Moreover, in *EB*_2_, *m* is divided by MMC washout rate, *µ*_1_. Using the estimated parameters of the model, this value is of 0.311[*µM*], implying that beyond 99.99% of the drug dose *m*_0_ was cleared, i.e., without side effects or toxicity. In other words, we suggest that this equilibrium point indicates cure only under the assumption that there remains a small amount, *M* ^∗^, of MMC in the bladder (under continuous therapy for a prolonged period of time).

Stability: The eigenvalues of the Jacobian at this equilibrium are: $\overset{¯}{\lambda }=\left[-\mu_{1},r-\frac{p_{2}d_{0}}{\mu_{2}}-\frac{p_{1}\frac{m}{\mu_{1}}}{\left(\frac{m}{\mu_{1}}+a\right)},-\mu_{2}\right]$. Therefore, all eigenvalues are negative, if:


(4) $$r<\frac{p_{2}d_{0}}{\mu_{2}}+\frac{p_{1}\frac{m}{\mu_{1}}}{\left(\frac{m}{\mu_{1}}+a\right)}.$$

The tumor-free (cure) equilibrium *EB*_2_ is locally asymptotically stable when *r< I*_1_ + *I*_2_ and unstable when *r > I*_1_ + *I*_2_.

A biological understanding of this criterion may be introduced as follows. At equilibrium, the first term on the right hand, *I*_1_, is the net daily killing rate of tumor by immune cells, as highlighted in condition (3). In the same way, the second term, *I*_2_, is the net daily killing rate of tumor by MMC chemotherapy:

1. *I*_2_ involves the MMC source, *m* molecules of drug that are introduced daily, multiplied by the killing rate of tumor cells via MMC effects, *p*_1_, to give the daily MMC efficacy.
2. This efficacy, *p*_1_ *m*, is limited by MMC decay, at a daily rate *µ*_1_, and the saturation effect given by the Michaelis-Menten form, with a half-saturation parameter, *a*.

Therefore, the right hand of (4) reflects the sum of all the killing effects of the tumor, by both the immune system and chemotherapy treatment. When this total killing rate is greater than the tumor growth rate *r*, the tumor-free equilibrium is stable (numerical simulation appears in **Supplementary Figure S4**). Should the tumor growth rate, *r*, be larger, the tumor’s strength governs the process and the equilibrium loses stability. The criterion shows the ranges for *r*, within which constant MMC treatment, together with effector cells (*E*), can clear the tumor to a state where, similar to *EB*_1_, the immune system is in homeostasis, free from side effects.

## 5 Results

We begin with formulating conditions for disease-free states through stability analysis. Subsequently, we compare the behavior of model simulations to clinical data, and conduct a numerical parameter sensitivity analysis on estimated parameters. Furthermore, we present an application in MMC dosage determination.

### 5.1 Mathematical conditions for tumor extinction

It is a main interest to identify the criteria for which tumor is cleared. Two equilibrium points in the current model exhibit the desired result of a healthy bladder, i.e., *T* = 0:

1. **Homeostasis:** When tumor is untreated, the body relies solely on its immune cells for defense. With respect to *EB*_1_, the homeostasis equilibrium, the stability criterion (4), to maintain the state of no tumor cells:


(3) $$r<\;\frac{p_{2}d_{0}}{\mu_{2}}.$$

2. **Tumor-free equilibrium:** A similar stability-criterion was found for *EB*_2_, the tumor-free equilibrium:


(4) $$r<\frac{p_{2}d_{0}}{\mu_{2}}+\frac{p_{1}\frac{m}{\mu_{1}}}{\left(\frac{m}{\mu_{1}}+a\right)}.$$

The competition of immune cells alone or in combination with chemotherapy against tumor cells is reflected in each criterion here by tumor killing and tumor forming activities, respectively. That is, as outlined in criterion (3), the destructive mechanisms of the immune system alone against tumor cells are strong enough to eliminate tumor cells only up to a certain threshold, *I*_1_, which is the upper bound for the tumor growth rate, *r*. This theoretical threshold increases in the criterion outlined by Equation (4), as MMC’s killing ability, *I*_2_, is added to the immune killing ability *I*_1_. As a result, the synchronization of all tumor killing effects enables to eliminate even more rapidly growing tumors, i.e., *r* ∈ (*I*_1_ *, I*_1_ + *I*_2_). For smaller destruction rates in each criterion, the tumor’s proliferation capacity dominates, resulting in the destabilization of *EB*_1_ and *EB*_2_.

### 5.2 Model examination

We are now interested in comparing the above model [system (1)] with data obtained from biological studies as well as previous mathematical models, as described below and in **Table 1** and **Supplementary Table S2**. Computer simulations were performed using fifth-order adaptive step Runge-Kutta integration, as implemented in the ode45 subroutine of MATLAB, to visualize approximations to the solution for the model ODEs. We also tested ode23s for systems with varying time scales involving cells and molecules. However, results showed no observable difference compared to the ode45 solver, indicating both are suitable for simulations. We start by illustrating distinct case scenarios of the disease as captured by our model. Then, we study the behavior of our model when confronted with oncological research.

Since no geometrical considerations are being taken in this paper, we used the term tumor size in the meaning of the number of tumor cells. To incorporate oncological terminology into the model, we followed the methodology outlined in (20) to translate the prognostic factor ‘tumor size’ for recurrence in BC (80) into tumor cell count. The tumor surface area was calculated assuming a circular shape and a 3-cells depth to determine the volume using the length of cell being approximately 10*µ*m. Given that 1mm^3^ ∼ 10^6^ cells (81), the formula of the number of tumor cells is:


(5) $$\#\text{Cells }=\pi {\left(\text{radius}\right)}^{2}h\times \;10^{6}\approx \pi \times \;{\left(\text{radius}\left[\text{mm}\right]\right)}^{2}\times \;3\;\times \;\left(10^{-2}\left[\text{mm}\right]\right)\;\times \frac{10^{6}\;\left[\text{cells}\right]}{1\;\left[\text{m}{\text{m}}^{3}\right]}$$.

The presence of recurring or residual tumors after TUR has been documented in the literature (39, 82). Therefore, we use the reasonable assumption that the diameter of each residual tumor is not more than the length of resection. Utilizing the formula given in Equation (5) we derive the corresponding initial tumor cells count, *T*(0), with regard to the initial clinical tumor sizes.

#### 5.2.1 Theoretical simulations

We simulated three types of scenarios according to the initial tumor cell count. The choices for tumor sizes based on the information that small BC tumors typically range from less than 20[mm] in diameter (83), with the lower limit of sensitivity for detecting BC tumors ranging from 5 to 10[mm] (84). Medium tumors are defined within the range of 20-50[mm], while large tumors are classified as *>* 50[mm] in the study of Loloi et al. (83):

1. The initial tumor cell count for a tumor of “small” size *T*(0) = 5.3 × 10^6^, corresponding to a size of 15[mm].
2. The initial tumor cell count for a tumor of “medium” size *T*(0) = 1 × 10^7^, corresponding to a size of 20.6[mm].
3. The initial tumor cell count for a “large” tumor *T*(0) = 6.62×10^7^, corresponding to a size of 26.5[mm]. This value is below the highest reported value of 75[mm], where successful tumor resections were performed, as noted in (85).

**Figure 2:**
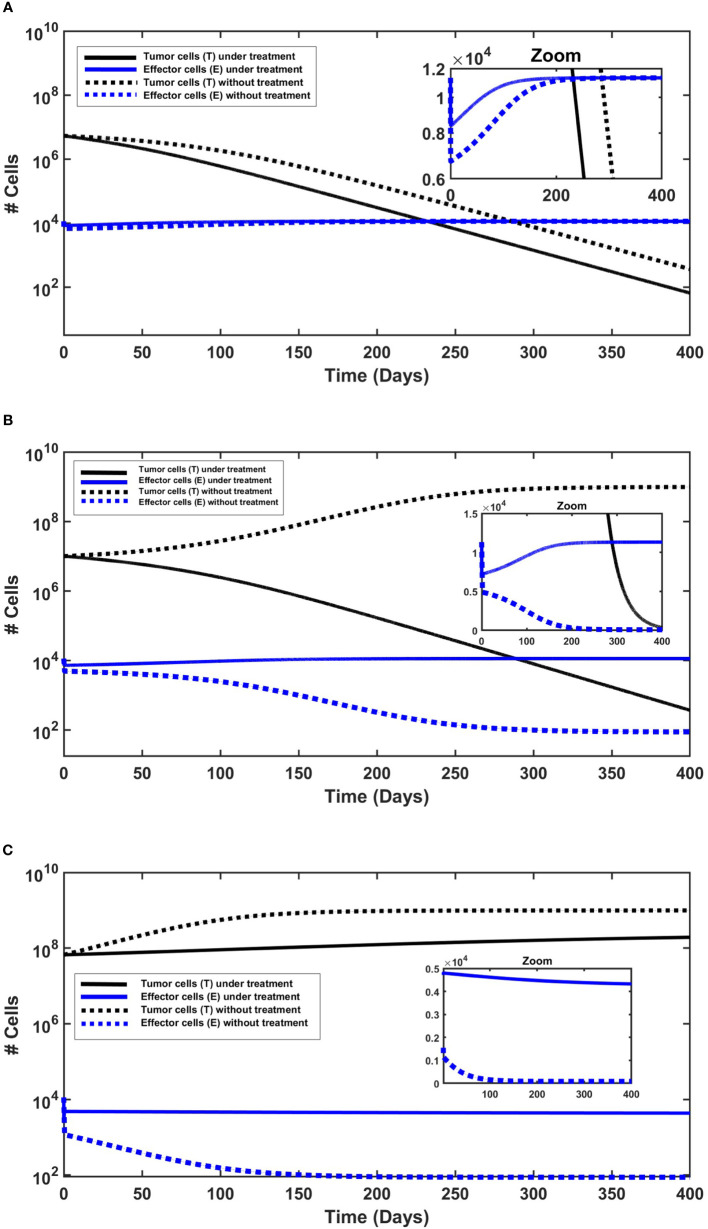
The behavior of urothelial tumor cells (black lines) and effector cells (blue lines), with (solid lines) and without (dashed lines) continued application of MMC. Initial conditions are found in the **Supplementary Information**. **(A)** Tumor elimination with and without chemotherapy. The elimination is slightly faster under continued application of MMC chemotherapy. **(B)** Cure only under chemotherapy. Without treatment there is a logistic growth of tumor cells (*T*), so there is no cure. **(C)** Tumor persistence with and without chemotherapy. Effector cells decrease, but more significantly without treatment.

In each numerical simulation, we showed the evolution of tumor in time with and without treatment. The simulated “small” tumors can be eliminated by the immune system (effector cells) only for initial tumor size of *T*(0)< 7.36 × 10^6^ (see **Figure 2A**), and if untreated, tumors with greater *T*(0) grow until they reach the carrying capacity. With the killing effects of MMC, as specified above, all treatment simulations resulted in decreased tumor cell counts (*T*), compared to the no-treatment scenario (see **Figures 2A-C**). However, MMC could eliminate “small” tumors for initial tumor size which is *T*(0) ≤ 2.93 × 10^7^. This suggests that MMC has a curative effect within a certain range of initial tumor cell numbers, which, for the results in **Figures 2A–C**, falls within 7.36 × 10^6^≤ *T*(0) ≤ 2.93 × 10^7^. If the number of cells exceeds a theoretical threshold for “large” tumors, the treatment will not be sufficient to cure BC (see **Figure 2C**). It is noteworthy that even when initial tumor sizes fall below this theoretical threshold, variations in model parameters, such as increased tumor growth rate *r* or decreased immune production rate *d*_0_, can result in a rise in tumor cell count, manifesting as tumor cell count beyond the theoretical threshold. To demonstrate this influence on tumor cell count, we conducted simulations with parameter variations (see Subsection 4.3 of the **Supplementary Information**).

The next step is to investigate the behavior of effector cells (*E*). In all simulations (**Figures 2A-C**), MMC treatment resulted in a higher effector cell counts compared to the untreated scenario. This is particularly evident in simulations with larger initial tumor sizes (**Figures 2B, C**), and subtly visible in **Figure 2A** due to the logarithmic scale of the y-axis; a zoom plot revealed a gap between treated and untreated scenarios, declining from about 1,700 cells after 1 day of simulation to approximately 50 cells only after 250 days. Studies of Hori et al. (54, 55) indeed suggest that immunosuppressive cells called regulatory T-cells (Tregs) which regulate effector cells counts, are reduced by MMC.

Regarding treatment outcomes, in cases where a cure is achieved, effector cell counts consistently increased throughout the entire simulated time (**Figures 2A, B**), approximately starting from the end of day 1 after the initial reduction. The initial reduction during the first day in all simulations (**Figures 2B, C**) reflects tumor burden (*p*_3_ *ET*) in Equation (1c), which, for given parameters and initial conditions, determines the sign of the term $\frac{dE}{dt}$ . Subsequently, a possible interpretation is that the dynamics are determined by the treatment outcome: an increase in effector cells over time if treatment is successful (*T* declines), or a decrease if tumor persistence occurs (*T* increases). Distinct behavior is shown in treatment failure (**Figure 2C**), showcasing an interesting case with a slight decrease in effector cell count from 1 day after treatment to the end point of the 400-day simulation. This can be explained by the larger initial tumor size, as all other initial conditions were identical to those in the other simulations. In other words, beyond Tregs regulation, tumor cells may indirectly induce a slight decrease in effector cells by triggering Tregs elevation beyond a specific threshold. Hori et al. (54, 55) do emphasize that cancer cells induce activation of Tregs, which play a role in controlling immune escape in cancer. The combined effects of MMC and the immune system’s killing effects appear insufficient for the complete eradication of tumor cells in this case.

#### 5.2.2 Qualitative comparison to oncological studies

The model is designed to find generic qualitative insights intrinsic to its structure. Accordingly, simulations should not be interpreted as predictions. In the absence of specific observed clinical data, we refer to our approach as ‘qualitative agreement’, based on the available observations and a comparison with literature consistency. We first confronted the model output to studies of NMIBC without MMC chemotherapy. In two studies (80, 83) that focused solely on TUR, larger-sized tumors were associated with a higher likelihood of developing postoperative complications and death. The model simulations without treatment show that indeed increasing the initial tumor size changed tumor dynamics from tumor elimination to tumor persistence (see **Figures 2A-C**). Under the model assumption of tumor size, our simulations demonstrate similar behavior: tumors that exhibit a cell count below 7.36 × 10^6^[cells] (*<* 17.68[mm]) are eliminated by the immune system, and for values above this threshold, no cure is evident.

In the assessment of treatment success, a step toward validation involves addressing the following inverse problem: given the prescribed dose of MMC in the reported protocol, what is the maximum threshold of initial tumor size, under the model for achieving a tumor-free state? To solve this problem, we varied the initial condition of tumor cell count, *T*(0), while keeping all other parameters and initial conditions unchanged (see calculations and simulations in the **Supplementary Information**). We used clinical research studies that analyzed the impact of a single MMC instillation post TUR surgery in patients with low-risk BC (tumor size of 30[mm] or less):

1. In the study by Solsona et al. (86), a 24-month follow-up revealed a significant increase in the recurrence-free interval, along with reduced recurrence and tumor rates per year in the MMC group compared to the control group. Our simulations, utilizing the study dosage, establish a threshold of 1.21×10^7^[cells], corresponding to a tumor diameter of 22.67[mm] (see **Supplementary Figure S7**).
2. The prospective study of Ersoy et al. (87), showed no recurrence of patients during the follow-up period of five years. Utilizing their dosage plan, the model attained a threshold of 2.14 × 10^7^[cells], corresponding to a diameter of 30.14[mm] (see **Supplementary Figure S8**).

Given that no tumor-immune specific parameters can be extracted from these studies, the model simulations can only hypothesize that a curative effect may extend to tumors up to a certain *x*[*mm*] size. That is, the resulting values only allow us to demonstrate the technical ability to calculate thresholds. However, simulations with treatment do demonstrate curative effects up to a diameter that exceeds those without treatment, thereby underscoring the positive impact of MMC compared to the control group.

In (86), recurrence was observed in some patients of study. This can be modeled by the inter-patient variability on the biological level- such as different tumor growth rate, different immune cells production rate, as shown in Subsection 4.3 of the **Supplementary Information**. We conclude that the obtained threshold values for cure state are dependent on the specific choice of the parameters which reflects a specific tumor-immune condition of the patient. This way we can resolve the slight changes in values from the range in these clinical studies.

### 5.3 Sensitivity analysis

**Figure 3:**
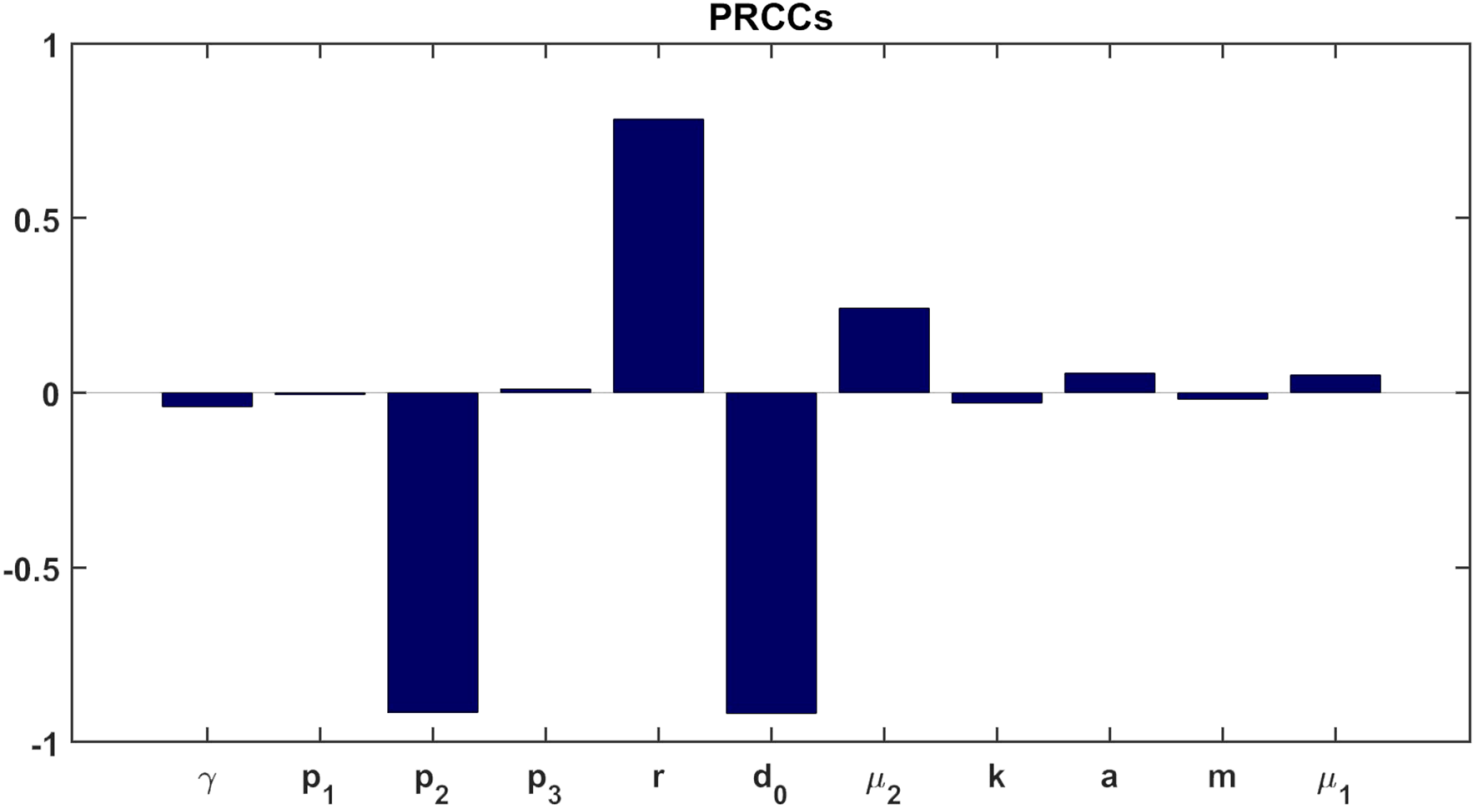
Statistically significant PRCC values (*p*-value< 0.01) for *T*(*t*) at day 365.

We conducted sensitivity analysis on all model parameters (see **Figure 3**). The analysis was performed with respect to the tumor cells count (*T*) at day 365 in the treatment case, which implies that at this specific time point, the total drug dosage administered to the hypothetical patient equals the dose of a single chemotherapy treatment (recall scaling of MMC dosage in Section 3). Following the methodology outlined by (88), we employed Latin hypercube sampling to generate 1000 samples, and chose the range of each parameter from 1*/*2 to twice its values in **Table 1**, adopting the strategy described in (89). These samples were then used to calculate the partial rank correlation coefficients (PRCC) and the *p*-values with respect to the tumor cells count (*T*) at day 365. We observe that parameters promoting anti-tumor dynamics, including the sources (input) parameters of chemotherapy and the immune system (*m*, *d*_0_), along with the killing and activation rates of the tumor by these elements (*p*_1_, *p*_2_, *γ*), demonstrate negative correlations with tumor size. Conversely, parameters that promote tumor growth and anti-tumor dynamics—the growth rate of the tumor (*r*), deactivation of immune cells (*p*_3_), mortality rate of immune cells (*µ*_2_), and chemotherapy washout rate (*µ*_1_)—exhibit positive correlations with tumor size. Overall, **Figure 3** reveals that the parameters *p*_2_ *, r*, and *d*_0_ have the greatest influence on tumor growth. In the quest for a deeper understanding of the influence of two parameters, *µ*_1_ and *a*, which can be estimated in various ways in the absence of empirical or consensus data, we conducted uncertainty analysis (see Section 6 in the **Supplementary Information**).

### 5.4 Application for MMC dose determination

Clinicians often consider ‘dosage determination’ as a critical aspect in improving MMC delivery and optimizing patient outcomes (39, 44). While variety of MMC chemotherapy treatment programs are available (90), new auxiliary tools are yet necessary to precisely determine the amount of MMC required to cure a specific BC patient.

To facilitate the process of personalized dose determination, we propose a new bio-mathematical algorithm of MMC for the treatment of BC patients. We use the following assumptions regarding the initial conditions of system (1), to describe patient state after MMC treatment:

- The completeness of tumor resection- during the TUR procedure almost all of tumor cells are removed, i.e., *T*(0) ≈ 0. Support for this assumption is found in the biological literature (16, 36, 91).
- The vast majority of MMC is cleared (92, 93), so that *M*(0) ≈ 0. Therefore, we can choose $\varepsilon =\frac{m}{\mu_{1}}$ which is negligible, such that *M*(0) = *ε*.
- There is a homeostatic production of immune cells, i.e., $E(0)\approx \frac{d_{0}}{\mu_{2}}$ . This assumption is reasonable since MMC is generally safe due to its limited effects to the local area of administration (18, 37).

This way, system (1) introduces small perturbations from the entries of the tumor-free equilibrium, *EB*_2_. As a next step, we will manipulate the expressions for stability criterion (4) of *EB*_2_:


(6) $$m<\;\frac{\mu_{1}a\;(\frac{p_{2}d_{0}}{\mu_{2}}-r)}{r-\;\frac{p_{2}d_{0}}{\mu_{2}}-\frac{p_{1}}{\mu_{1}}}.$$

To ensure that the condition in Equation (6) is biologically valid for MMC instillation rate, *m*, it is essential to verify that the right hand of (6) is positive. We obtain the condition given by Equation (7):


(7) $$\frac{p_{2}d_{0}}{\mu_{2}}<r<\;\frac{p_{2}d_{0}}{\mu_{2}}+\;\frac{p_{1}}{\mu_{1}}.$$

**Figure 4:**
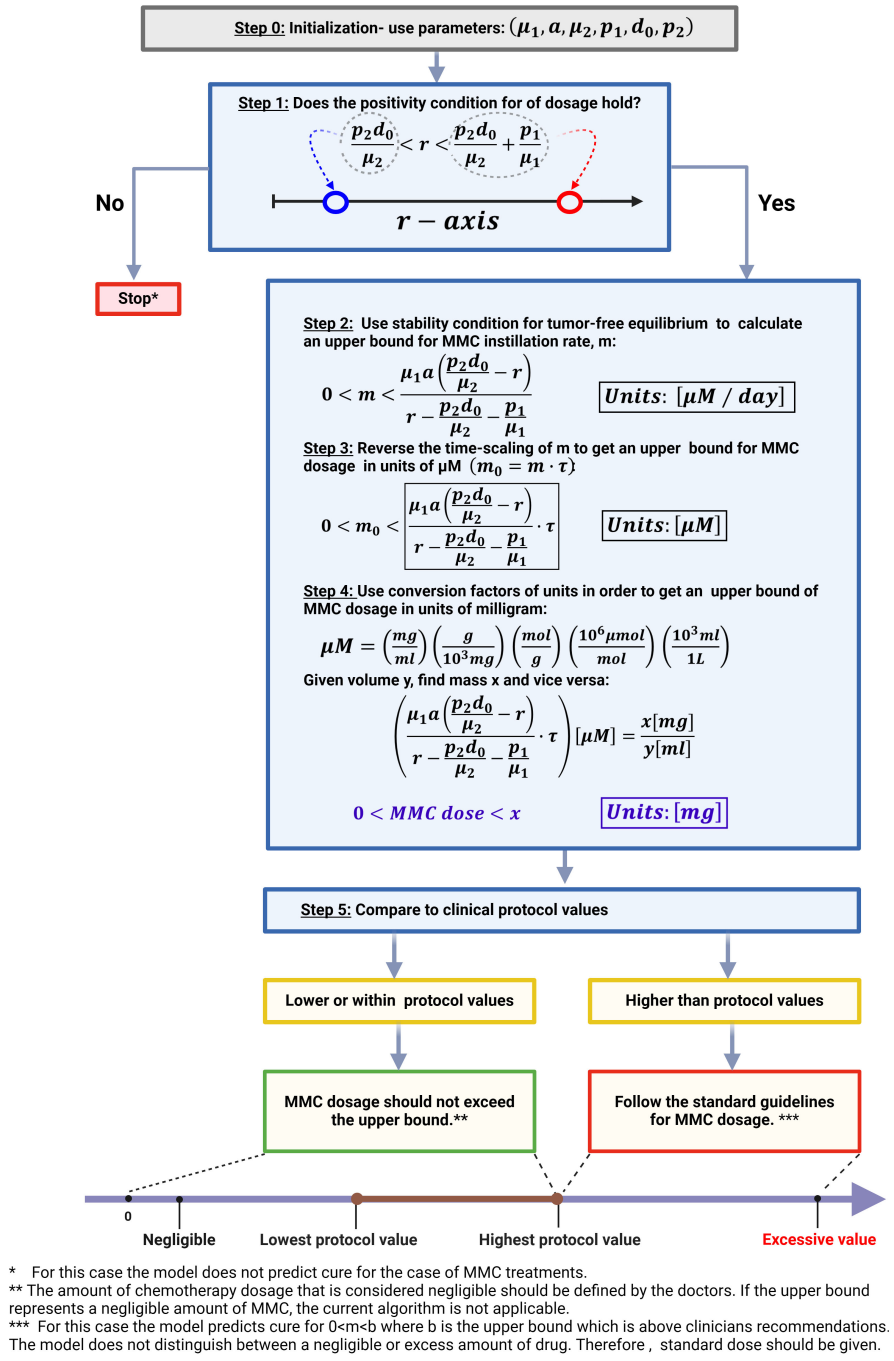
Algorithm for MMC drug dose determination in treating BC patients. This image was created with BioRender.com.

Note that the range for *r* is valid only if both the numerator and denominator of the right-hand side of criterion (6) are negative. Utilizing local stability condition for *EB*_2_, the dynamical system suggests cure. The obtained criterion (6) enables the calculation of an upper bound for the MMC dose at which treatment is successful, based on theoretically patient-specific parameters. A step-by-step description of the algorithm can be found in **Figure 4**. The algorithm categorizes patients into two groups; One group for theoretical patients eligible for dose calculation—where the model facilitates determining a dose below the clinically recommended maximum. This indicates that the model identifies a dosage interval within which a cure, according to the model’s criteria, is achieved. Should the upper bound surpass the recommended maximum, we interpret this as over-toxicity, emphasizing the significance of considering the recommended range. It is important to note that, in this particular case, the recommended range is within the curative range. Yet, for enhancing the specificity of the curative range, future efforts will require a focus on determining a lower bound for the curative range, in addition to restricting it by the upper bound. The second group, labeled as ‘theoretical non-responders’, consists of patients excluded due to theoretically specific parameters not meeting the cure conditions (6) − (7) or receiving an insignificantly small calculated dose.

**Figure 5:**
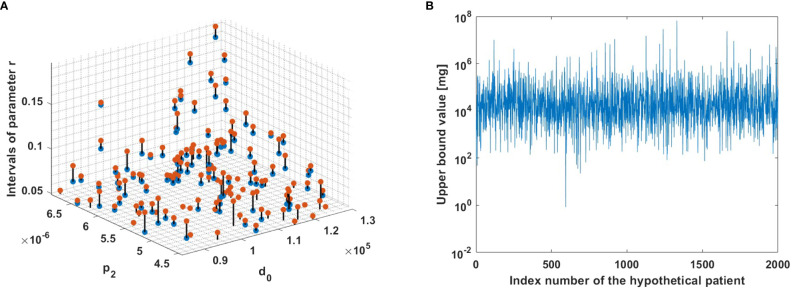
The model algorithm applied for 2,000 hypothetical patients. **(A)** Ranges for parameters *r*, such that *m* is positive. The blue and red dots correspond to the left and right interval endpoints in (7), respectively. The observed intervals are depicted in the figure using a zoom-in view to enhance visualization. **(B)** Upper bounds for MMC dosage. The upper bound for each hypothetical patient was computed using criterion (6).

To demonstrate the robustness of the method for a large number of patients in a 3-D plot, we adopted a systematic approach through virtual experiments with variations of all parameters except r which is determined by the algorithm. The algorithm was used for 2,000 different hypothetical patients to understand how parameters affect the performance of the method (see **Figure 5**). Variations of parameters from **Table 1** were performed, but such that *I*_1_ < *r* holds. This choice enabled us to exclude instances where parameter sets do not provide positivity of MMC dosage. Thus, the focus was on cases for which a feasible MMC dosage could be calculated; excluded cases are viewed as theoretical non-responders, i.e., further investigation is required beyond the scope of this paper. For each patient, criteria (6)−(7) provided a range of growth rates *r* for which MMC dosage is positive. For each one of the calculated intervals of *r* values, one value was chosen randomly by the Matlab rand function, and for this value an upper bound for therapeutic MMC dosage was calculated in units of *mg*. It is noteworthy to observe that intervals of *r* with higher values are observed alongside higher values of *d*_0_ and *p*_2_.

## 6 Discussion

Arguments regarding MMC’s role in curing NMIBC have been made in many articles (56, 58, 75). This study adds a quantitative basis to these considerations, by showing that under the model assumptions, the complex biological processes of NMIBC, the immune system, and MMC interactions can be captured by a relatively simple 3-dimensional system. The model exhibits four non-negative equilibrium points, which depend on chemical and biological related parameters. The nontrivial dependence of the dynamics on tumor growth (*r*) is emphasized as oncologically relevant. Particularly, the model analysis in biological context suggested the following distinct dynamical patterns, which are explicitly dependent upon the appropriate range of *r* (see **Supplementary Figures S1-S4**). Under no treatment, *m* = 0, homeostatic phase of a healthy bladder is conserved for low tumor formation rates *r < I*_1_, meaning that the immune system alone is capable of clearing bladder tumor cells. The tumor is eradicated exponentially fast as immune activity *I*_1_ increases. Violation of this condition results in a logistic expansion of the tumor. When MMC is administered, *m >* 0, it is important to note that stability of tumor-free equilibrium, means that as soon as MMC chemotherapy is added, the killing effects of chemotherapy are added to the process, enabling the elimination of tumors with a faster growth rate than the homeostatic phase, *r < I*_1_ + *I*_2_. For high tumor formation rates *r > I*_1_ + *I*_2_, in view of the limited killing effects of the immune system and MMC, it is impossible to cure tumors that proliferate very rapidly.

In light of the increasing prominence of mathematical models in cancer research (7), our model did add new elements into the current discussion on treating BC patients, as well as detecting hypothetical properties that are not evident in experimental studies. Chemotherapy protocol investigation is one of these features. Based on a distinctive viewpoint of local stability, we designed a new method to calculate an upper bound to the drug dose, *m*_0_, for which chemotherapy is successful. At its current stage, this method remains theoretical, offering valuable insights into the structural dynamics of the model and its capacity to accommodate unit conversion factors relevant to drug measurements via stability analysis. The analytical procedure allows to classify patients into two groups given a theoretically specific set of patient’s parameters: those who will benefit from the treatment and those who will not. When the upper bound of MMC amount cannot be calculated or is negligible, the model output does not suggest cure. Otherwise, it is possible to determine the individual drug dosage as shown in **Figure 4**. We concluded that this kind of range is clinically relevant because it may potentially reveal the effectiveness of the treatment protocol in a nontrivial way compared to classic pharmacological studies which are PK/PD-model-based (9). Furthermore, only a limited number of clinical MMC dose comparison studies have been conducted. For example, in (94), the researchers performed a prospective, randomized study to compare 30 mg and 40 mg of MMC dose for BC patients. This approach is realistic since it provides the actual biological results. However, due to the impracticality of testing all possible dosing options in clinical trials, the efficacy of these studies is inherently constrained.

The clinical observation that low-risk bladder cancer (BC) patients form an inhomogeneous group, that can be stratified by tumor size (95), is highlighted in the model through simulations (**Figures 2A-C**). The simulations suggest that chemotherapy success depends on the initial tumor size, but also on model parameters (see **Figure 3**). As described in the literature (59), our simulations demonstrate the effectiveness of MMC that in some cases offers a better cure, and even provide a theoretical threshold of tumor cells number that can be eliminated, given a hypothetical specific set of parameters.

To enable the results above to help doctors evaluate the risks of MMC treatment protocols for BC patients individually, it is essential to measure specific clinical parameters for each one of them. In other words, the presented parameter values and ranges are not asserted as the single possible choice; rather, they serve to demonstrate the adaptability of incorporating biological data into modeling. The eventual refinement of all model parameters will be dictated solely by future findings in biological research. To begin with, the assessment of the tumor growth characteristics in patients *in vivo* to calibrate the growth rate, *r*. Currently, we are not aware of any *in-vivo* method to continuously track the value for this parameter. With regard to the measurement of the MMC’s properties in the human bladder, it is necessary to investigate the specific washout rate *µ*_1_ and killing capacities of MMC for calibration of the killing parameter *p*_1_ and the Michaelis-Menten half-saturation constant *a*. One step in this endeavor involves the establishment of a bio-bank comprising patient-derived BC organoids for the assessment of drug responses (96). Similarly, progress has been made with the examination and count of tumor infiltrating lymphocytes (TIL) in biopsy samples (97). However, immune cells (CTLs and DCs) production and death rates- *d*_0_ *, µ*_2_, respectively, are yet to be found in thorough measurements supported by extensive datasets.

Current model was created to formulate explicit mathematical relations, such that a balance between analytical tractability and biological credibility was maintained. However, this study has limitation by its exclusion of the spatial structure of tumors. Existing models recognize the significance of incorporating the spatial structure of tumors for obtaining realistic results (98–100). Another limitation is exclusion of the heterogeneity within tumor and immune cell populations; specific subpopulations like BC stem cells and Tregs are acknowledged in biological literature for their association with resistance to MMC treatments (54, 56). For instance, the autophagy process, known to be involved in BC resistance to MMC, is associated with BC stem cells (56). Furthermore, the lack of characterization of specific behaviors in immune cell subpopulations, such as the regulatory role of Tregs in tumor dynamics (63, 101), may lead to potential misconceptions about immune system mechanisms. This is evident in the assumption of constant effector cell production (*d*_0_). It does not include important aspects of effector cell proliferation, where DCs play a vital role in adaptive immunity by presenting antigens and activating T cells. Activated DCs can migrate to lymph nodes to prime naïve T cells (63), while Tregs regulate these immune responses. These processes significantly influence effector cell dynamics. Toward an in-depth understanding of the underlying biology, future work should address these limitations.

Finally, MMC is currently considered one of the most effective chemotherapy treatments after TUR to prevent NMIBC. In this paper, we propose theoretical explanation for the fact that still, a substantial percentage of patients fail the treatment (38–43). It is possible that treatment failure results from tumor size, as described in the literature (86, 87) and shown in **Figure 5**, or from an improper selection of the drug dose, *m*_0_, that is implicitly reflected in the drug instillation rate, *m*, of the model. In this context, improper is defined as a set of parameters that are theoretically patient-specific and do not meet the conditions required by this mathematical method for cure. In particular, as outlined in the sensitivity analysis section, and as evident from the illustrated behavior in **Figure 3**, for higher values of parameters *p*_2_ and *d*_0_, in some cases yield higher values of growth rate *r* such that MMC dosage could be calculated. However, this does not guarantee a valid upper bound for dosage. Upon proper extensions and a thorough validation, the model can potentially pave the way for developing predictive tools for BC growth and determining curative drug dosages.

## Data availability statement

Data and code to run model simulations and generate all figures are available at https://github.com/MYAUni/MMC-Model. Further inquiries can be directed to the corresponding author.

## Author contributions

MY: Visualization, Writing – original draft, Writing – review & editing, Formal analysis, Software, Data curation. SB-M: Conceptualization, Project administration, Supervision, Funding acquisition, Writing – review & editing.
