# Supplementary material for: Mathematical model of MMC chemotherapy for non-invasive bladder cancer treatment
Source: Front Oncol. 2024 May 31;14:1352065. doi: 10.3389/fonc.2024.1352065 (PMC11176538; doi:10.3389/fonc.2024.1352065)
Supplement: Supplementary file 1 [file DataSheet_1.pdf]

Supplementary Material

1 INVARIANCE OF POSITIVE ORTHANT

THEOREM 1. *Let $T(t)$, the tumor cell count, be bounded by the carrying capacity, $0 \leq T(t) \leq k$ for all t. Then, trajectories of model equations (1) remain in the positive orthant $\mathbb{R}^3_+$ for all time, i.e. the region is invariant.*

PROOF. Assume that the model begins with positive initial conditions (all parameters are positive):

$$M(0) > 0, \quad T(0) > 0, \quad E(0) > 0.$$

We use some elementary considerations.

- $M(t) > 0$ for all $t \in [0, +\infty]$ if $M(0) > 0$.
  Given that the initial condition is $M(0)$ we get the following:

$$\frac{dM}{dt} = -\mu_1 M + m$$
$$\frac{dM}{dt} = -\mu_1 \left(M - \frac{m}{\mu_1}\right)$$

  We divide by $\left(M - \frac{m}{\mu_1}\right)$ which is nonzero (otherwise, we get the solution $M(t) = M(0) > 0$ for all $t \in [0, +\infty]$, and the proof is complete for this case), to solve:

$$\frac{\frac{dM}{dt}}{M - \frac{m}{\mu_1}} = -\mu_1$$
$$\int \frac{dM}{M - \frac{m}{\mu_1}} = -\mu_1 \int \mathrm{d}t$$
$$\log\left(M - \frac{m}{\mu_1}\right) = -\mu_1 t + c$$
$$M - \frac{m}{\mu_1} = ce^{-\mu_1 t}$$

  Then,

$$M(t) = ce^{-\mu_1 t} + \frac{m}{\mu_1}$$

  Determining the constant c by substituting $t = 0$ in $M(t)$ we get:

$$M(0) = c + \frac{m}{\mu_1} \Rightarrow c = M(0) - \frac{m}{\mu_1} \Rightarrow M(t) = \frac{m}{\mu_1}\left(1 - e^{-\mu_1 t}\right) + M(0)e^{-\mu_1 t}.$$

  The general solution of the MMC equation $(1a)$ is given by: $M(t) = \frac{m}{\mu_1}\left(1 - e^{-\mu_1 t}\right) + M(0)e^{-\mu_1 t}$. Since $e^{-\mu_1 t} \leq 1$ for all $t \in [0, +\infty]$, then $\left(1 - e^{-\mu_1 t}\right) \geq 0$. As m, μ_1 are positive, we get that the first

term in the solution is non-negative. For the second term, non-negativity follows from $e^{-\mu_1 t} > 1$ and $M(0) > 0$. Therefore, $M(t) > 0$ for all $t \in [0, +\infty]$ given that $M(0) > 0$.

- $T(t) > 0$ for all $t \in [0, +\infty]$ if $T(0) > 0$.
  From $0 \leq T \leq k$ it follow that $rT\left(1 - \frac{T}{k}\right) \geq 0$. Then, $\frac{dT}{dt} \geq -T\left(\frac{p_1 M}{M+a} + p_2 E\right)$. Hence, T is more than or equal to the solution of $\frac{dT}{dt} = -T\left(\frac{p_1 M}{M+a} + p_2 E\right)$. Let $T(0) = T_0$. Then,

$$T(t) \geq T_0 \exp\left(-\int_0^t \left[\frac{p_1 M}{M+a} + p_2 E\right] \mathrm{d}t\right) > 0 \text{ for all } t \in [0, +\infty] \text{ if } T_0 > 0$$

- Similar to the proof for T, we prove $E(t) > 0$ for all $t \in [0, +\infty]$ if $E(0) > 0$.
  Given that $M(t), T(t)$ are nonnegative for all $t \in [0, +\infty]$ and that d_0, γ, p_1, a are positive parameters, we get $d_0 + \gamma \frac{p_1 TM}{M+a} \geq 0$. Then $\frac{dE}{dt} \geq -E\left(p_3 T + \mu_2\right)$. Hence, $E(t)$ is more than or equal to the solution of $\frac{dE}{dt} = -E\left(p_3 T + \mu_2\right)$. Let $E(0) = E_0$. Then,

$$E(t) \geq E_0 \exp\left(-\int_0^t \left[p_3 T + \mu_2\right] \mathrm{d}t\right) > 0 \text{ for all } t \in [0, +\infty] \text{ if } E_0 > 0$$

Thus, the positive orthant $\mathbb{R}^3_+$ is invariant and for all t: $M(t) > 0, T(t) > 0, E(t) > 0$. □

This property is used for stability analysis.

2 INITIAL CONDITIONS

Table S1. List of initial conditions for the model.

Initial condition	Value	Source
$M(0)$	0	Assumption
$T(0)$	0	Bunimovich-Mendrazitsky et al. (2007)
$E(0)$	$\left[0, 1.36 \times 10^7\right]$ cells	Calculation from sources below, Bunimovich-Mendrazitsky et al. (2011)

We choose the following initial conditions (summarized in **Table S1**):

- MMC, $M(0)$:
  We assumed that the drug is given only via the input parameter m, i.e., $M(0) = 0$.
- Tumor cells, $T(0)$:
  The carrying capacity of tumor cells, k, was estimation is $1 \times 10^9 [cells]$. Therefore, we take $T(0) \in [0, 10^9]$.
- Effector cells, $E(0)$:
  In Bowyer et al. (2022), a rough estimation indicated that DCs subsets constituted approximately 20% of the bladder immune cells. An analysis estimate for the total count of immune cells in the bladder is approximately 10^9 (Sender et al., 2023). We make the simplifying assumption that the ratio between bladder tumor weight and total bladder weight is proportional to the ratio between their respective cell counts. Using this assumption, we derive the following formula to obtain a rough estimate of $E(0)$:

$$\# \text{ DCs } = \#(\text{ total number of bladder immune cells }) \times \frac{\text{bladder tumor weight}}{\text{bladder weight}} \times \text{ fraction of DCs} \quad \text{(S1)}$$

Considering the median tumor resection weight reported as $1.4g$ (Hald et al., 2023) and $2g$ (Fernández-Conejo et al., 2020), with the overall estimated weight falling within the range of $(29.3 - 42)g$, we derive the highest theoretical value for initial effector cell counts:

$$E(0) = 10^9 \times \frac{2}{29.3} \times 0.2 = 1.36 \times 10^7,$$
$$\text{Therefore } E(0) \in \left[0, 1.36 \times 10^7\right].$$

The specific values of initial conditions and parameters for all model simulations are stated below in **Table S2**.

Table S2. List of the model initial conditions and parameters. Here ε is a perturbation, $\varepsilon = 10$.

	**Initial condition** $(M(0), T(0), E(0))$	**Parameters set**
**Figure 2(a)**	$\left(0, 4 \times 10^6, d_0/\mu_2\right)$	$p_1 = 0.17, r = 0.032$
**Figure 2(b)**	$\left(0, 1 \times 10^7, d_0/\mu_2\right)$	$p_1 = 0.17, r = 0.032$
**Figure 2(c)**	$\left(0, 8 \times 10^7, d_0/\mu_2\right)$	$p_1 = 0.17, r = 0.032$
**Figure 3**	$\left(0, 1 \times 10^8, d_0/\mu_2\right)$	$p_1 = 0.17, r = 0.032$
**Figure 5**	The algorithm exclusively utilizes parameters	$p_1 = 0.17, r = 0.032$
**Figure S1**	$(\varepsilon^*, \varepsilon^*, d_0/\mu_2 + \varepsilon^*)$.	$r = 0.01, p_2 = 5 \times 10^{-6}$
**Figure S2**	$\left(\varepsilon^*, 1.066 \times 10^7 + \varepsilon^*, 3957.338 + \varepsilon^*\right)$	$r = 0.02, p_2 = 5 \times 10^{-6}$
**Figure S3**	$\left(\varepsilon^*, 9.835 \times 10^8 + \varepsilon^*, 65.605 + \varepsilon^*\right)$	$r = 0.02, p_2 = 5 \times 10^{-6}$
**Figure S4**	$(m/\mu_1 + \varepsilon, 0 + \varepsilon, d_0/\mu_2 + \varepsilon)$	$r = 0.02, p_2 = 5 \times 10^{-6}$
**Figure S5**	$\left(0, 1 \times 10^8, d_0/\mu_2\right)$	$p_1 = 0.12, p_2 = 0.37 \times 10^{-5}$, $p_3 = 1.59 \times 10^{-6}, d_0 = 8.256 \times 10^4$, $r = 0.045$
**Figure S6(a)**	Multiple-found in text	$p_1 = 0.17, r = 0.032$
**Figure S6(b)**	Multiple-found in text	$p_1 = 0.17, r = 0.032$
**Figure S6(c)**	Multiple-found in text	$p_1 = 0.17, r = 0.032$
**Figure S7**	Multiple-found in text	$p_1 = 0.12, r = 0.032$
**Figure S8**	Multiple-found in text	$p_1 = 0.12, r = 0.032$
**Figure S9**	$\left(0, 1 \times 10^8, d_0/\mu_2\right)$	$p_1 = 0.17, r = 0.032$

3 STABILITY ANALYSIS FOR THE MODEL

3.1 Steady states derivation

The equilibria of the model are found by setting all derivatives to zero and solving for M^*, T^* and E^*, with the star notation indicating the variables are at their equilibrium values. For convenience, disease-free equilibrium points are assigned the indexing of EB, and cancer equilibrium points are assigned the indexing of C. The model has multiple equilibria but, given the invariance of the positive orthant, we need only focus on the nonnegative equilibria assuming all initial conditions are positive:

- $\frac{dM}{dt} = -\mu_1 M + m,$
  Here, the parameter m denotes two states of chemotherapy administration that are described separately in two distinct case scenario later on:
  1. $m = 0$ in the absence of chemotherapy instillation;
  2. $m > 0$ for constant chemotherapy instillation.

Setting all derivatives to zero, we get the system:

$$\begin{cases} -\mu_1 M + m = 0, \\ -T\left(\frac{p_1 M}{M+a} + p_2 E\right) + rT\left(1 - \frac{T}{k}\right) = 0 \\ d_0 - E\left(p_3 T + \mu_2\right) + \gamma \frac{p_1 TM}{M+a} = 0. \end{cases} \tag{S2}$$

We'll consider two cases using the solution of the first equation of (S2).

3.1.1 No treatment is given, i.e, $m = 0$, so $M^* = 0$:

$$\begin{cases} -p_2 TE + rT\left(1 - \frac{T}{k}\right) = 0 \Longrightarrow T_1^* = 0, T_2^* = \dfrac{k}{r}\left(r - p_2 E\right), \\ d_0 - E\left(p_3 T + \mu_2\right) = 0. \end{cases} \tag{S3}$$

- **For $\mathbf{T_1^* = 0}$:**
$$d_0 - \mu_2 E = 0 \Longrightarrow E^* = \frac{d_0}{\mu_2}. \tag{S4}$$
  Thus, the first equilibrium is: $EB_1 = (M^*, T^*, E^*) = \left(0, 0, \dfrac{d_0}{\mu_2}\right)$.

- **For $\mathbf{T_2^* = \frac{k}{r}\left(r - p_2 E\right)}$:**
$$\frac{p_2 p_3 k}{r} E^2 + \left(-p_3 k - \mu_2\right) E + d_0 = 0. \tag{S5}$$
  $\Rightarrow E_{2,3}^* = \frac{\rho \pm \sqrt{\rho^2 - 4\xi d_0}}{2\xi}$. where $\rho = p_3 k + \mu_2, \xi = \frac{p_2 p_3 k}{r}$. First, to get real solutions we demand $\Delta \geq 0$, i.e., $\rho^2 - 4\xi d_0 \geq 0$, implying that $\rho \geq 2\sqrt{d_0 \xi}$ or $\rho \leq -2\sqrt{d_0 \xi}$. Now since $\rho = p_3 k + \mu_2 > 0$ (parameters are positive), solution are real when $\rho \geq 2\sqrt{d_0 \xi}$ holds. From positivity of parameters $\sqrt{\rho^2 - 4\xi d_0} < \sqrt{\rho^2} = \rho$., i.e., $E_{2,3}$ is positive (assuming that the solutions are real). So, when $\rho > 2\sqrt{d_0 \xi}$ it follows that $E_{2,3} \geq 0$, and we get two more equilibrium points:
$$C_{1,2} = (M^*, T^*, E^*) = \left(0, k - \frac{p_2 k}{r}\left(\frac{\rho \pm \sqrt{\rho^2 - 4\xi d_0}}{2\xi}\right), \frac{\rho \pm \sqrt{\rho^2 - 4\xi d_0}}{2\xi}\right).$$

3.1.2 Treatment is given $m > 0$:

$$\begin{cases} -\mu_1 M + m = 0 \Longrightarrow M^* = \frac{m}{\mu_1}, \\ -T\left(\frac{p_1\left(\frac{m}{\mu_1}\right)}{\left(\frac{m}{\mu_1}\right)+a} + p_2 E\right) + rT\left(1-\frac{T}{k}\right) = 0, \Longrightarrow T^* = 0, T^* = k - \frac{k}{r}\left(\frac{p_1\left(\frac{m}{\mu_1}\right)}{\left(\frac{m}{\mu_1}\right)+a} + p_2 E\right) \\ d_0 - E\left(p_3 T + \mu_2\right) + \gamma\frac{\beta p_1 T\left(\frac{m}{\mu_1}\right)}{\left(\frac{m}{\mu_1}\right)+a} = 0. \end{cases} \tag{S6}$$

For the second equation in $(S6)$ to be zero, we have two options:

- **First case:** $T^* = 0$:

$$d_0 - \mu_2 E = 0 \tag{S7}$$
$$\Longrightarrow E^* = \frac{d_0}{\mu_2}.$$

  The corresponding equilibrium is: $EB_4 = (M^*, T^*, E^*) = \left(\frac{m}{\mu_1}, 0, \frac{d_0}{\mu_2}\right)$.

- **Second case:** $T^* = k - \frac{k}{r}\left(\frac{p_1\left(\frac{m}{\mu_1}\right)}{\left(\frac{m}{\mu_1}\right)+a} + p_2 E\right)$. Substitution in the third equation of (S6) :

$$d_0 - E\left(p_3\left(k - \frac{k}{r}\left(\frac{p_1\left(\frac{m}{\mu_1}\right)}{\left(\frac{m}{\mu_1}\right)+a} + p_2 E\right)\right) + \mu_2\right) + \gamma\beta p_1 m \frac{\left(k - \frac{k}{r}\left(\frac{p_1\left(\frac{m}{\mu_1}\right)}{\left(\frac{m}{\mu_1}\right)+a} + p_2 E\right)\right)}{\mu_1\left(\frac{m}{\mu_1}+a\right)} = 0. \tag{S8}$$

$$\Rightarrow E^*_{2,3} = \frac{-\varphi \pm \sqrt{\varphi^2 - 4\psi\omega}}{2\psi}$$

Where $\psi = \frac{p_2 p_3 k}{r}, \varphi = -p_3 k - \mu_2 + \frac{p_1 p_3 k\left(\frac{m}{\mu_1}\right)}{r\left(\left(\frac{m}{\mu_1}\right)+a\right)} - \frac{p_1 p_2 k m \gamma\beta}{r\mu_1\left(\left(\frac{m}{\mu_1}\right)+a\right)}, \omega = d_0 + \frac{p_1 k m \gamma\beta}{\mu_1\left(\left(\frac{m}{\mu_1}\right)+a\right)} - \frac{p_1^2 m^2 \gamma\beta k}{r\mu_1^2\left(\left(\frac{m}{\mu_1}\right)+a\right)^2}$

Accordingly, the substitution produces cumbersome calculations for this quadratic equation in variable E. In the absence of a trivial biological interpretation for the parametric form of the equilibria, we used Maplesoft to calculate them numerically after insertion of parameters and initial conditions from Table 2 and Table **S1** (expecting at most two points):

$$C_3 = (M^*, T^*, E^*) = (1365.463, -258033.024, -18361.260),$$
$$C_4 = (M^*, T^*, E^*) = \left(1365.463, -1.649 \times 10^{11}, 641355.583\right).$$

For C_3 the number of tumor cells (T) and effector cells (E) is negative. Similarly, for C_4, the number tumor cells (T) is negative. These are valid mathematical results, with no biological meaning. Hence, we don't examine.

Overall, in the absence of treatment, we obtained three equilibrium points in the model (system $(S2)$), and in the presence of treatment, we obtained one equilibrium point from system $(S6)$.

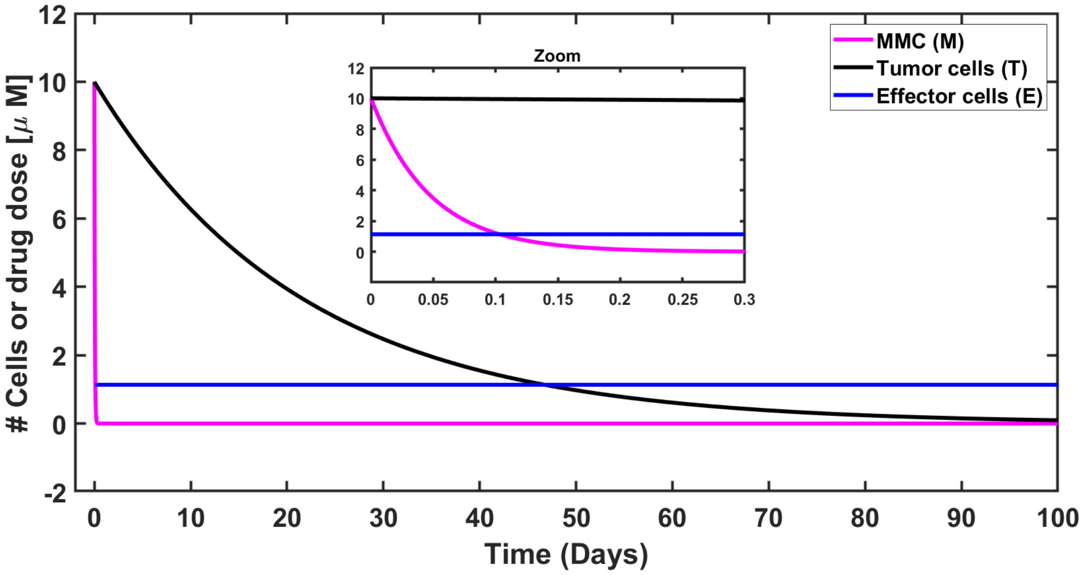

Figure S1. Homeostasis equilibrium EB_1^*. Notice, that the scale for effector cells (E) was divided into 10^4 for the possibility to show all curves in one graph. Tumor cells (**black** solid line) and MMC dose (**magenta** solid line) decrease during the estimated period (up to 100 days), while effector cells (**blue** solid line) remain constant starting from few hours after the disturbance, as they go to $d_0/\mu_2 = 1.032 \times 10^5/9.12 = 11,315.789$ (which is 1.131 in this graph due to scaling). In this case scenario, the individual will be eventually healthy.

3.2 Simulations of stability analysis

The behavior of urothelial tumor cells (T), effector cells (E) , and MMC dose (M) (**black**, blue, and **magenta** solid curves, respectively) for each equilibrium point is depicted; in Figures S1 and S4 for the disease-free equilibrium points $EB_{1,2}$, and in Figures S2 and S3 for the cancer equilibrium points $C_{1,2}$, respectively. To display all curves in each graph, we used scaling (see Table S1).

We present the following investigation of $C_{1,2}$:

$$C_{1,2} = \left(0, k - \frac{p_2 k}{r}\left(\frac{\rho \pm \sqrt{\rho^2 - 4\xi d_0}}{2\xi}\right), \frac{\rho \pm \sqrt{\rho^2 - 4\xi d_0}}{2\xi}\right), \text{ where } \rho = p_3 k + \mu_2, \xi = \frac{p_2 p_3 k}{r}$$

The points here corresponds to a state where the tumor and immune cells are both present (non-negativity condition for their values is provided in the Supplementary Information). The process of finding stability criteria for $C_{1,2}$ involves cumbersome calculations. In the absence of a trivial biological interpretation for the parametric form of the equilibria, the complexity of these stability conditions may hinder a straightforward interpretation of the system's behavior. Therefore, we used substitution of parameters to determine stability:
- $C_1 = (M^*, T^*, E^*) = \left(0, 1.066 \times 10^7, 3957.338\right)$, which is the "small" tumor equilibrium, with Jacobian So we get $\bar{\lambda} = [-21.049, -26.09, 0.012]$. Therefore, the fixed point obtained by insertion it into Jacobian is not stable (see Figure S2). - The other solution is the "large" tumor equilibrium: $C_2 = (M^*, T^*, E^*) = \left(0, 9.835 \times 10^8, 65.605\right)$. From the obtained eigenvalues $\bar{\lambda} = \left[-21.050, -1573.042, -1.928 \times 10^{-2}\right]$, it follows that EB_3 is locally asymptotically stable, as all eigenvalues are negative (see Figure S3).

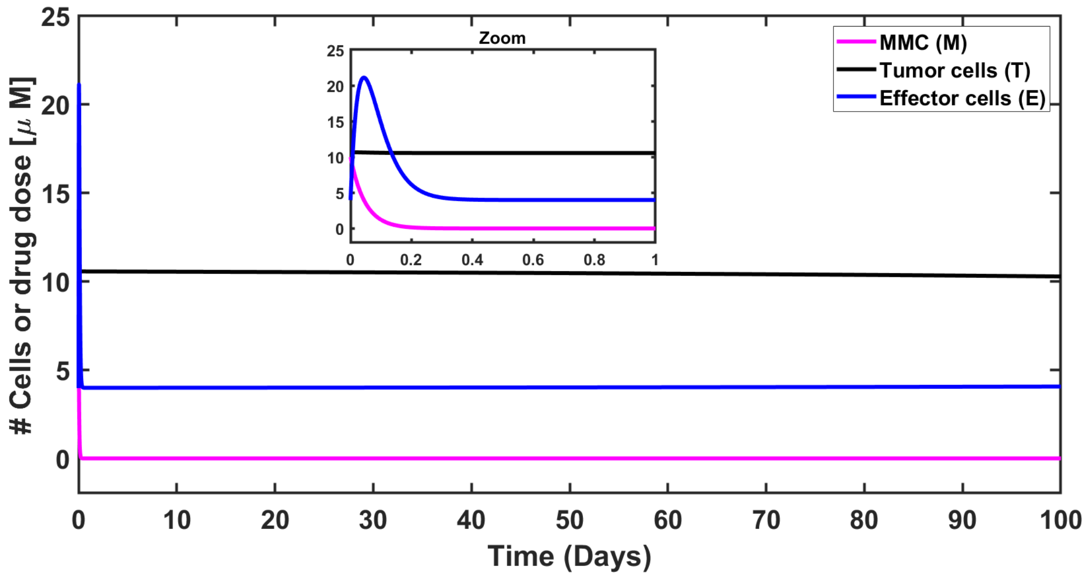

Figure S2. The small tumor equilibrium C_1^*. Notice, that the scale for tumor cells (T) was divided into 10^6, and for effector cells (E) by 10^3, for the possibility to show all curves in one graph. We can see that tumor cells (**black** solid line) and MMC dose (**magenta** solid line) decrease during all estimated time. Effector cells (blue solid line) fluctuate during first day and then increase during all estimated time (up to 100 days). Hence, we see that the variables are changing in time and eventually diverge from the small tumor equilibrium (not stable at all).

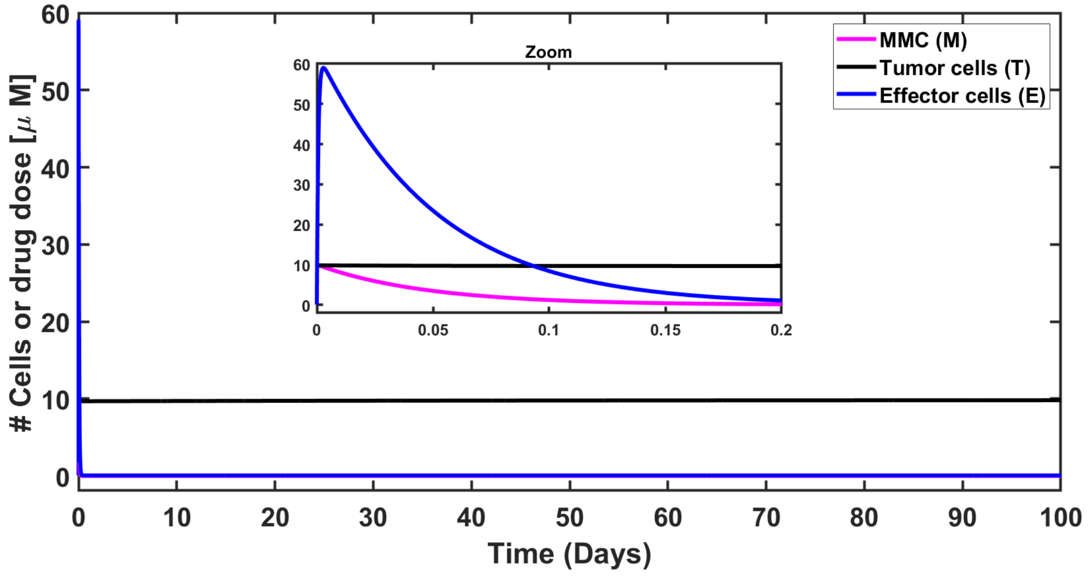

Figure S3. The large tumor equilibrium C_2^*. Notice, that the scale for tumor cells (**black** solid line) was divided into 10^8 and effector cells (**blue** solid line) by 10^3, for the possibility to show all curves in one graph, starting from the zoom graph (note that during the first day both effector cells and drug dosage decline drasically). We can see that during the first hours, both effector cells and MMC decline drastically. In less than one day, all variables stay unchanged during almost all estimated time (up to 100 days), i.e., a large tumor persistence.

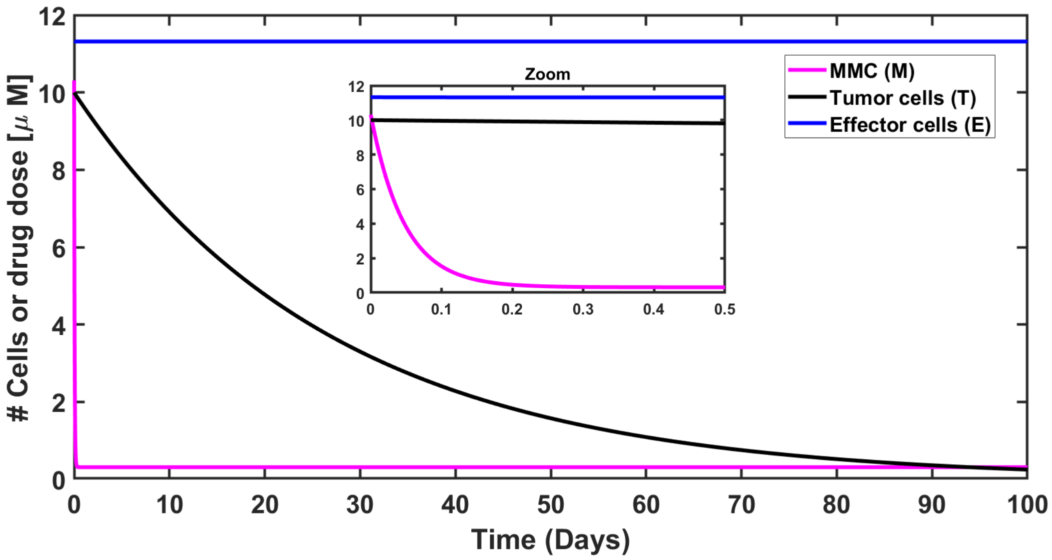

Figure S4. Tumor-free equilibrium EB_2^*. Notice, that the scale for effector cells (E) was divided into 10^3 for the possibility to show all curves in one graph. We can see that after 100 days tumor cells (**black** solid line) declines to zero while effector cells (**blue** solid line) and MMC dose (**magenta** solid line) stay stable with no change during all estimated time (up to 150 days). We chose parameters so that they satisfy the stability condition above.

4 EVALUATION OF CENTRAL MODEL ASSUMPTIONS

4.1 Rationale for constant effector cells production

One aspect of the model's structure involves the assumption of constant effector cell production, dictated by the constant parameter d_0. In the following sections, we elaborate on the rationale behind this assumption.

4.1.1 Qualitative perspective

- DCs reside in the bladder tissue in homeostatic (Bowyer et al., 2022).
- The review conducted by Merad et al. (2009) indicates that tissue-DC homeostasis requires constant replacement with new cells. Therefore, the decision to model constant production arises from the natural turnover of antigen-presenting cells (APCs), particularly dendritic cells (DCs), in the bladder, as previously observed and modeled for other diseases (Marino and Kirschner, 2004), including non-muscle-invasive bladder cancer (NMIBC) (Bunimovich-Mendrazitsky et al., 2011). Consequently, we represented the source of effector cells (E) using d_0, providing an inclusive value, given that CTLs are activated by DCs (Hori et al., 2017, 2019), and that the model mentioned above in Bunimovich-Mendrazitsky et al. (2011) has modeled CTL counts proportional to the APC count.

4.1.2 Quantitative perspective

We utilize reported values and frequencies from biological sources to assess whether the model's terms and structure can accommodate values consistent with those observed in the bladder. The model assumes continuous replacement of 1.032×10^5 $[cells]$ per day, based on the reported replenishment rate of DCs (Liu et al., 2007). The aim is to evaluate if this term adequately describes counts reported in both homeostatic and BC states, employing two different estimations.

The model represents homeostasis with the term $\frac{d_0}{\mu_2}$, representing the ratio of immune production to mortality rate. This term's value is calculated as $11,316$ cells. It's essential to note that our goal is to ensure the correct order of magnitude for these parameters. However, given the absence of continuous counts of long time periods of NMIBC data, calibration is not currently feasible. Therefore, our assessment focuses on the qualitative agreement of our chosen parameters with the homeostatic effector cells count, constituting a fundamental evaluation:

- The absolute counts of total live DCs for healthy and tumor groups in orthotopic model of BC showed approximately $10^4 - 10^5$ cells, and CD8+ cells are of order of magnitude of $10^4 - 10^5$ for tumor group and 10^2 for healthy bladder (Senserrich et al., 2022). Therefore, taking this value, for the term effector cells the incompasses DCs and CTLs takes values which is within the observed order (also for selecting $\frac{d_0}{\mu_2}$ as the initial condition).
- DCs reside in the bladder tissue in homeostatic phase. Using the information given in Bowyer et al. (2022) that healthy murine bladders contain $30,000 - 50,000$ $\mathrm{CD45}^+$ cells, and DCs are the most prevalent immune cell in the bladder. They account for around 25% of all $\mathrm{CD45}^+$cells: $7,500 - 12,500$ DCs. The model value is within this range.

The choice of this parameter is based on biological sources suggesting consistency with the order of magnitude of the homeostatic and BC immune cell count. Furthermore, theoretical drug dose determination and simulations in following sections explore variations in this parameter, ensuring comprehensive analysis.

4.2 Rationale for scaled MMC instillation rate

In constructing our model, we considered that dividing the treatment dose, m_0, by 365 days might yield a linear drug response, contrary to the nonlinear patterns observed in oncological studies of MMC for NMIBC (Zhu et al., 2013; Shi et al., 2018). To address this concern, we incorporated the nonlinear Michaleis-Menten term $\frac{p_1 M}{M+a}$ into the equations governing tumor cells (T) and effector cells (E) in system (1), introducing saturation behavior to the model. Additionally, sensitivity analysis revealed that m is not among the most influential parameters (see Subsection 5.3). Furthermore, we explored the potential indirect effects of administering a single large dose. If the response were linear, a single large MMC dose would be expected to induce a massive elimination event of tumor cells and a substantial recruitment of immune cells, thereby shifting the tumor into rapid control mode—a scenario not achievable with continuous low doses. To assess this, we conducted numerical simulations simulating a single large MMC dose at ten times the dose m_0 (from which we obtain the instillation rate, m).

Although we observe an increase in immune cells in Figure S5, the maximal value seen is approximately 2×10^5, which decreases to 1.1×10^4 at the end of 400 days. This aligns with our estimated values for immune cell counts from biological sources in Table S2. However, no rapid tumor cell elimination is observed, and the decline is spread over the 400-day period, with tumor cell counts remaining above 1×10^6. Consequently, the observed curves support the validity of our approach in scaling the drug instillation rate.

4.3 Distinct simulation scenarios based on parameters variations

We aim to elucidate the profound impact of three key parameters - d_0, r, and p_2 - which, according to our sensitivity analysis detailed in Subsection 5.3, hold significant influence on tumor size. Considering a medium-sized tumor with an initial count of $T(0) = 1 \times 10^7$, variations in each of these parameters induce transitions between disease states. Specifically, alterations in the growth rate parameter r (illustrated in Figure S6a) dictate tumor growth intensity, while adjustments in immune system parameters such as

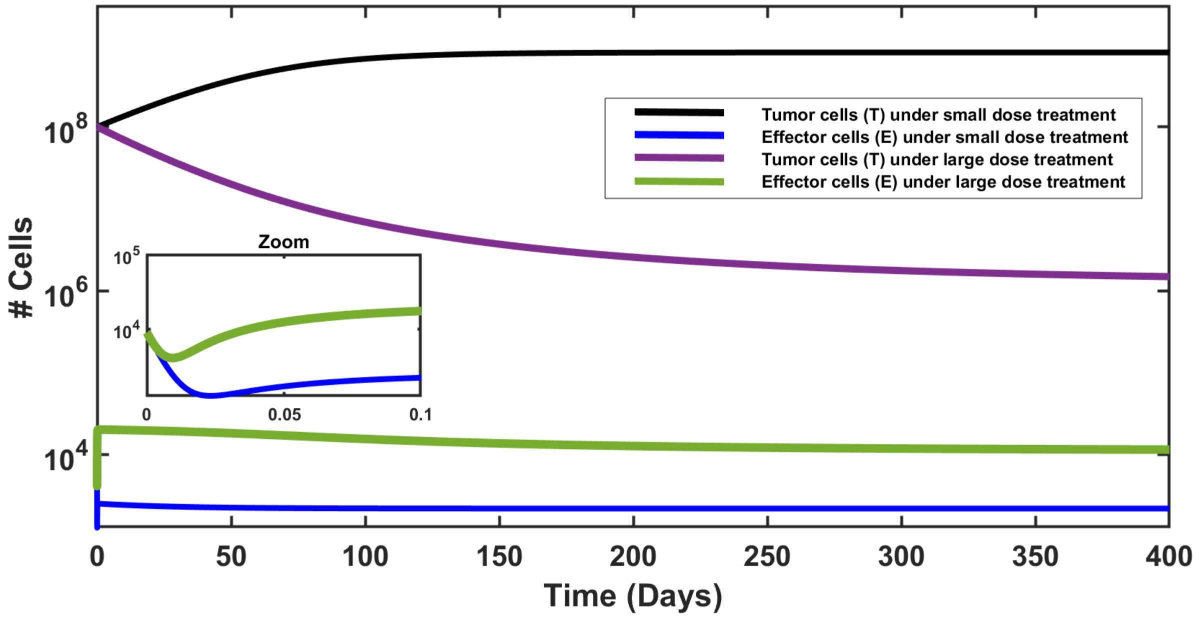

Figure S5. Comparison of simulation results for large $(10 \times m_0)$ **and small dose** (m_0) **treatments.**

the higher killing parameter p_2 or production rate d_0 (depicted in Figures $S6$ b and $S6c$) influence the strength of the immune response. That is, the changes in parameter values also govern shifts in disease states, alongside initial tumor size.

4.4 Qualitative agreement with oncological studies

In two studies the outcomes of a single MMC instillation, with results classified by tumor size are described (Solsona et al., 1999; Ersoy et al., 2013). This aligns with the same type of treatment that the model was built to describe. The qualitative examination was done by translating drug dose in the studies protocolos into parameters, from units of $[mg/ml]$ to $[\mu M]$:

- In Solsona et al. (1999), patients in the standard treatment group received 30 mg of MMC diluted with 50 mL of sterile saline (simulated in Figure $S7$):

$$m_0 = (\text{ Protocol dose }) \times (\text{ Mw of MMC }) = \left(\frac{30\text{mg}}{50\text{ml}}\right)\left(\frac{\text{g}}{10^3\text{mg}}\right)\left(\frac{1\text{ mol}}{334\text{ g}}\right)\left(\frac{10^6\mu\text{mol}}{1\text{ mol}}\right) = 1.796[\mu\text{mol/ml}] = 1,796[\mu\text{M}].$$

  The corresponding drug instillation rate, m:
  $m = \frac{m_0}{\tau} = \frac{1{,}796[\mu M]}{365\text{ days}} = 4.92[\mu M/day]$.

- In Ersoy et al. (2013), patients in the standard treatment group received 40 mg of MMC diluted with 40 mL of sterile saline (simulated in Figure $S8$)::

$$m_0 = (\text{ Protocol dose }) \times (\text{ Mw of MMC }) = \left(\frac{40\text{mg}}{40\text{ml}}\right)\left(\frac{\text{g}}{10^3\text{mg}}\right)\left(\frac{1\text{ mol}}{334\text{ g}}\right)\left(\frac{10^6\mu\text{mol}}{1\text{ mol}}\right) = 2.994[\mu\text{mol/ml}] = 2,994[\mu\text{M}].$$

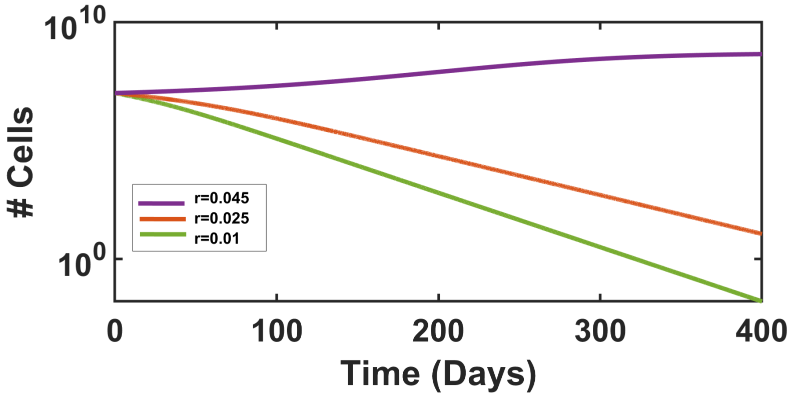

Figure 6a. Variations in r.

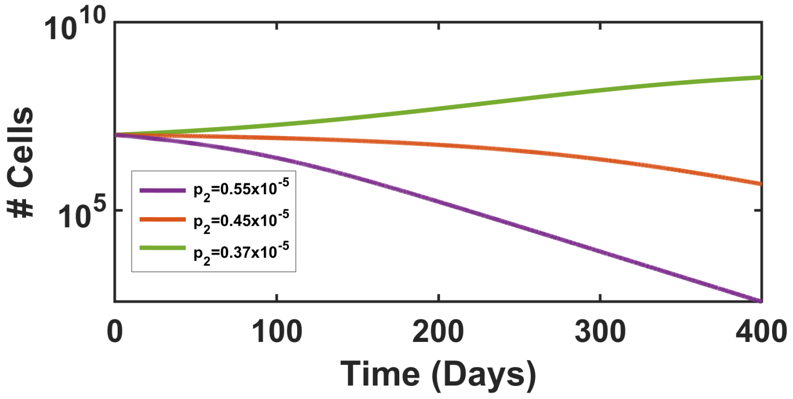

Figure 6b. Variations in p_2.

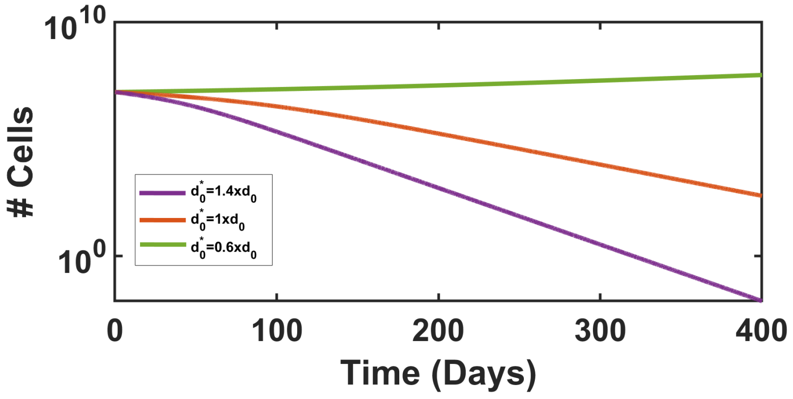

Figure 6c. Variations in d_0. d_0^* is the notation for the varied d_0.

Figure 6. Tumor size (T) curves for single parameter variations in each simulation.

The corresponding drug instillation rate, m:
$m = \frac{m_0}{\tau} = \frac{2{,}994[\mu M]}{365 \text{ days}} = 8.202[\mu M/day]$.

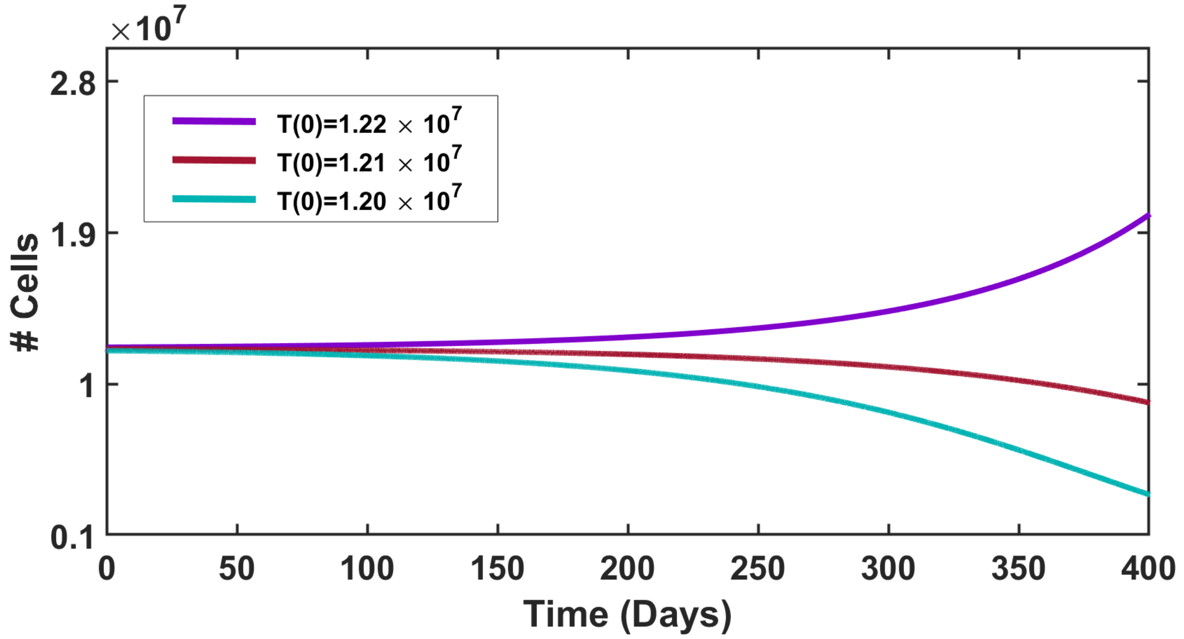

Figure S7. Threshold for $T(0)$ with the dose administration of Solsona et al. (1999). Here, we used the following initial conditions for M and E: $M(0) = 0$ and $E(0) = d_0/\mu_2$.

The simulated results to determine the highest value of $T(0)$ for successful treatment are presented in Figures $S7$ and $S8$. We regard cure as decrease in tumor cell count (for longer periods, the simulations show that this number declines below 1). In both cases, we utilized a minimum value for the MMC killing rate, $0.12[day^{-1}]$, to exclude results that may not be universally applicable to all patients, given the varied effectiveness of the drug among different individuals. Crucially, it must be clarified that the continuous administration of MMC aims to simplify the treatment process, typically delivered over a few hours. It unequivocally does not imply an intention for ongoing drug administration beyond the recommended clinical duration, or to predict treatment outcomes.

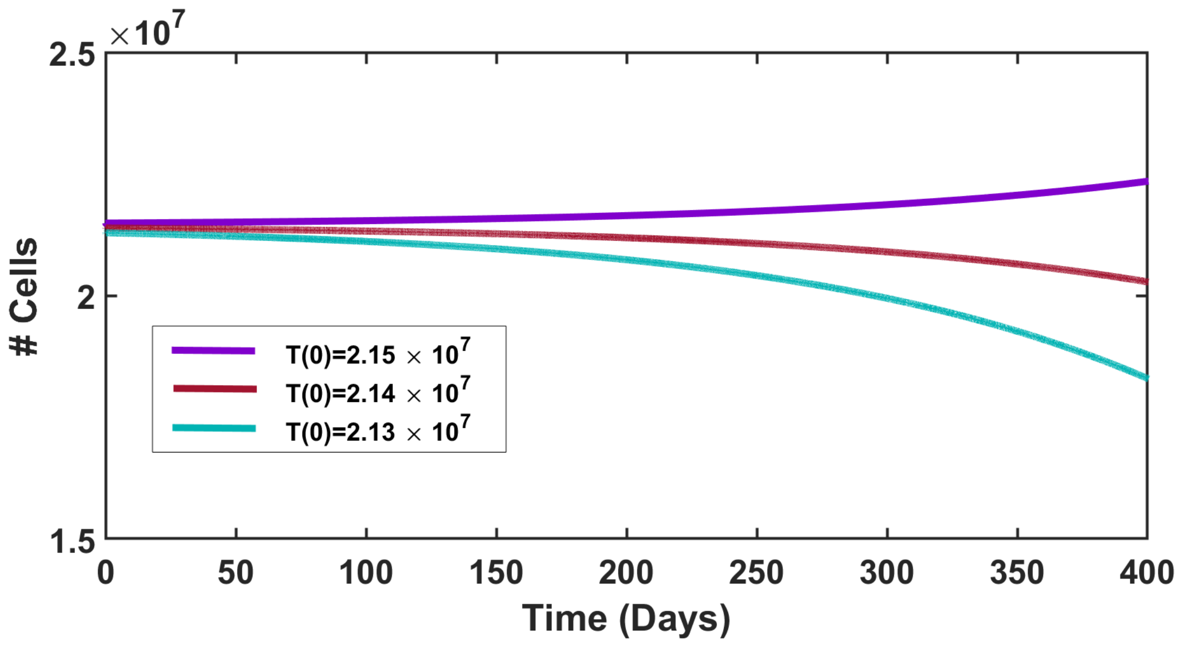

Figure S8. Threshold for $T(0)$ with the dose administration of Ersoy et al. (2013). Here, we used the following initial conditions for M and E: $M(0) = 0$ and $E(0) = d_0/\mu_2$.

5 DRUG DOSE DETERMINATION

For example, a direct calculation in the same manner as in each hypothetical patient case:

1. **Step 0- Use theoretical parameters set:**

$$(\mu_1, a, \mu_2, p_1, d_0, p_2) = (21.05, 100, 9.12, 0.12, 10^5, 1.82296 \times 10^{-6})$$

2. **Step 1- Verify positivity condition (7) for m:** the range for the growth rate r of which (7) holds:

$$0.0199 < r < 0.0256.$$

3. **Step 2- Solve condition (6) for m:** To substitute $r = 0.02$ that was chosen arbitrarily from the range found in step 1:

$$0 < m < 4.219[\mu\text{M}/\text{ day }].$$

4. **Step 3- Reverse time scaling of m:** To multiply by $365[\text{day}]$ to get the dosage m_0 in units of μM (see detailed calculation in step 3, Figure 4):

$$m_0 < 1,539.935[\mu\text{M}].$$

5. **Step 4- Use conversion factors to translate $[\mu\text{M}]$ unit into *mg* :** To find x, the mass in mg of the upper bound of MMC dosage, simply use the representation of the drug dose in $[\mu\text{M}]$ units as a product conversion factors,with only the value for *mg* unknown (see step 4, Figure 4) :

$$1,539.935[\mu M] = \left(\frac{x \text{ mg}}{50 \text{ ml}}\right)\left(\frac{\text{g}}{10^3 \text{ mg}}\right)\left(\frac{1\text{mol}}{334 \text{ g}}\right)\left(\frac{10^6\mu\text{mol}}{1 \text{ mol}}\right)\left(\frac{10^3 \text{ ml}}{1L}\right)$$

$$\implies x = 25.716.$$

Equivalently, the sterile water volume for a given mass of MMC can be determined.

6. **Step 5- Compare to the literature:** This result is within the range of treatment protocols. The EAU guidelines recommended $20 - 40\text{mg}$ as the standard MMC dose (Logan et al., 2012; Racioppi et al., 2019). A reasonable dose combining the model results and the clinical recommendation is $(20 - 25.716)$mg. Therefore:

$$\textbf{MMC dose} = 20 - 25.716\text{mg (in 50ml sterile water)}.$$

6 UNCERTAINTY ANALYSIS OF ESTIMATED PARAMETERS

Uncertainty analysis was performed with respect to the tumor cells count (T) for the parameters μ_1 and a at day 365. We selected these parameters because they can be estimated in numerous ways in the absence of empirical or consensus data. We took the range of each parameter from half to twice its value in **Table 1**. Figure S9 presents the different dynamics of the system for the tumor population count. The x-axis is the values of parameter μ_1 in units of $[\mu M]$, while the y-axis is the values of parameter a in units of $\left[day^{-1}\right]$. The color column shows cells population count, providing a simplified interpretation of tumor size, for any pair of (μ_1, a); the maximum number of tumor cells is 8.041×10^8. We see that T is an increasing function of each of the parameters μ_1, a alone and combined. The reason is the following: if μ_1 is small then the time for MMC to be washed out is longer, and if a is small then the amount of MMC required for the tumor-killing reaction rate to reach half of its maximum is small. Therefore, a smaller value of both parameters could result in a more favorable situation. With regard to μ_1, it was indeed reported that the clinical efficacy of intravesical MMC chemotherapy is constrained by the rapid clearance (Joice et al., 2019). The variation in tumor cell counts here may be viewed as a result of different patient conditions. Through conducting this analysis for μ_1 and a, we aim to underscore the importance of further biological research to determine their values.

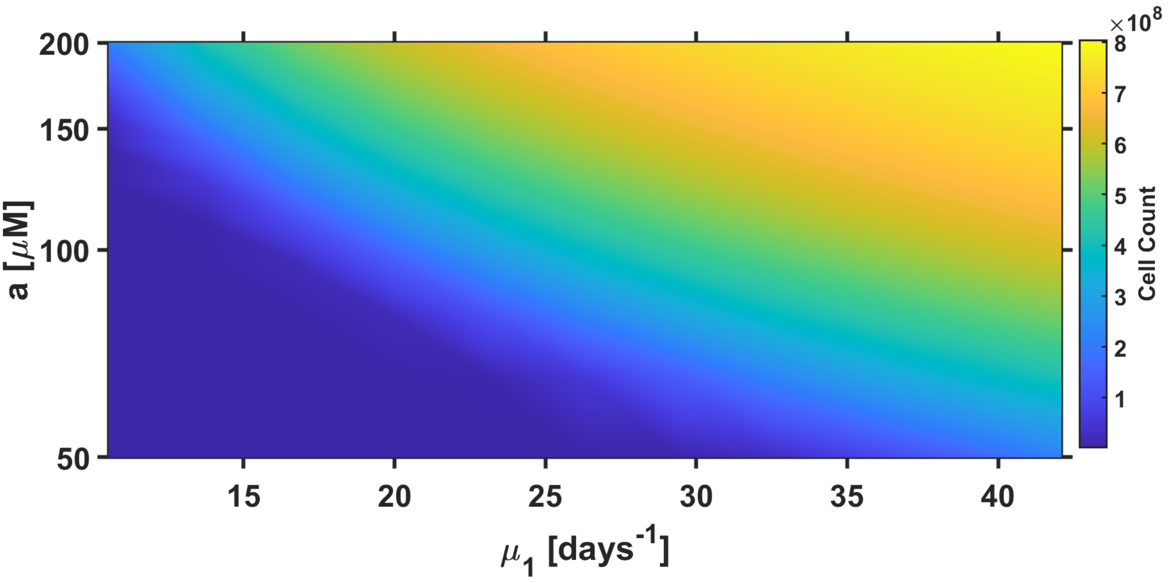

Figure S9. The color column shows the tumor cells count $T(\mu_1, a)$ when μ_1 varies between $(10.525 - 42.1)$ $\left[day^{-1}\right]$ and a between $(50 - 200)$ $[\mu M]$. All other parameter values remain the same as in Table 1.

REFERENCES

Bunimovich-Mendrazitsky S, Shochat E, Stone L. Mathematical Model of BCG Immunotherapy in Superficial Bladder Cancer. *Bulletin of mathematical biology* **69** (2007) 1847–70. doi:10.1007/s11538-007-9195-z.

Bunimovich-Mendrazitsky S, Claude Gluckman J, Chaskalovic J. A mathematical model of combined bacillus calmette-guerin (BCG) and interleukin (IL)-2 immunotherapy of superficial bladder cancer. *Journal of Theoretical Biology* **277** (2011) 27–40. doi:https://doi.org/10.1016/j.jtbi.2011.02.008.

Bowyer GS, Loudon KW, Suchanek O, Clatworthy MR. Tissue immunity in the bladder. *Annual Review of Immunology* **40** (2022) 499–523. doi:10.1146/annurev-immunol-101220-032117. PMID: 35471839.

Sender R, Weiss Y, Navon Y, Milo I, Azulay N, Keren L, et al. The total mass, number, and distribution of immune cells in the human body. *Proceedings of the National Academy of Sciences* **120** (2023) e2308511120. doi:10.1073/pnas.2308511120.

Hald OC, Phan YC, Carter CJ, Klatte T. Bladder tumor resection weight as a prognostic factor for recurrence and progression in patients with high-risk non-muscle invasive bladder treated with BCG. *Clinical Genitourinary Cancer* **21** (2023) e70–e77. doi:https://doi.org/10.1016/j.clgc.2022.08.007.

Fernández-Conejo G, de la Peña E, Hernández V, Guijarro A, Castro A, Pérez-Fernández E, et al. The value of tumour weight as a predictive factor for recurrence and progression in non-muscle invasive bladder cancer. *Scandinavian Journal of Urology* **54** (2020) 40–45. doi:10.1080/21681805.2019.1708968. PMID: 31905033.

Merad, Miriam, Manz, G M. Dendritic cell homeostasis. *Blood* **113** (2009) 3418–3427. doi:10.1182/blood-2008-12-180646.

Marino S, Kirschner DE. The human immune response to mycobacterium tuberculosis in lung and lymph node. *Journal of Theoretical Biology* **227** (2004) 463–486. doi:https://doi.org/10.1016/j.jtbi.2003.11.023.

Hori S, Miyake M, Tatsumi Y, Onishi S, Morizawa Y, Nakai Y, et al. Topical and systemic immunoreaction triggered by intravesical chemotherapy in an N-butyl-N-(4-hydroxybutyl) nitorosamine induced bladder cancer mouse model. *PLOS ONE* **12** (2017). doi:10.1371/journal.pone.0175494.

Hori S, Miyake M, Tatsumi Y, Morizawa Y, Nakai Y, Onishi S, et al. Intravesical treatment of chemotherapeutic agents sensitizes bacillus Calmette-Guerin by the modulation of the tumor immune environment. *Oncology Reports* **41** (2019). doi:10.3892/or.2019.6965.

Liu K, Waskow C, Liu X, Yao K, Hoh J, Nussenzweig M. Origin of dendritic cells in peripheral lymphoid organs of mice. *Nature immunology* **8** (2007) 578–83. doi:10.1038/ni1462.

Senserrich J, Guallar-Garrido S, Gomez-Mora E, Urrea V, Clotet B, Julián E, et al. Remodeling the bladder tumor immune microenvironment by mycobacterial species with changes in their cell envelope composition. *Frontiers in Immunology* **13** (2022). doi:10.3389/fimmu.2022.993401.

Solsona E, Iborra I, Ricós JV, Monrós JL, Casanova J, Dumont R. Effectiveness of a single immediate mitomycin C instillation in patients with low risk superficial bladder cancer: short and long-term followup. *The Journal of Urology* **161** (1999) 1120–1123. doi:https://doi.org/10.1016/S0022-5347(01)61606-9.

Ersoy H, Yaytokgil M, Karakoyunlu AN, Hikmet Topaloglu LS, Ozok HU. Single early instillation of mitomycin C and urinary alkalinization in low-risk non-muscle-invasive bladder cancer: a preliminary study. *Drug Design, Development and Therapy* **7** (2013) 1–6. doi:10.2147/DDDT.S39541.

Logan C, Brown M, Hayne D. Intravesical therapies for bladder cancer – indications and limitations. *BJU International* **110** (2012) 12–21. doi:https://doi.org/10.1111/j.1464-410X.2012.11619.x.

Racioppi M, Di Gianfrancesco L, Ragonese M, Palermo G, Sacco E, Bassi P. Chemoablation with intensive intravesical Mitomycin C treatment: A new approach for non–muscle-invasive bladder cancer. *European Urology Oncology* **2** (2019) 576–583. doi:https://doi.org/10.1016/j.euo.2018.08.032.

Joice G, Bivalacqua T, Kates M. Optimizing pharmacokinetics of intravesical chemotherapy for bladder cancer. *Nature Reviews Urology* **16** (2019). doi:10.1038/s41585-019-0220-4.
