# Supplementary figures and images for: Mathematical model of MMC chemotherapy for non-invasive bladder cancer treatment
Source: Front Oncol. 2024 May 31;14:1352065. doi: 10.3389/fonc.2024.1352065 (PMC11176538; doi:10.3389/fonc.2024.1352065)

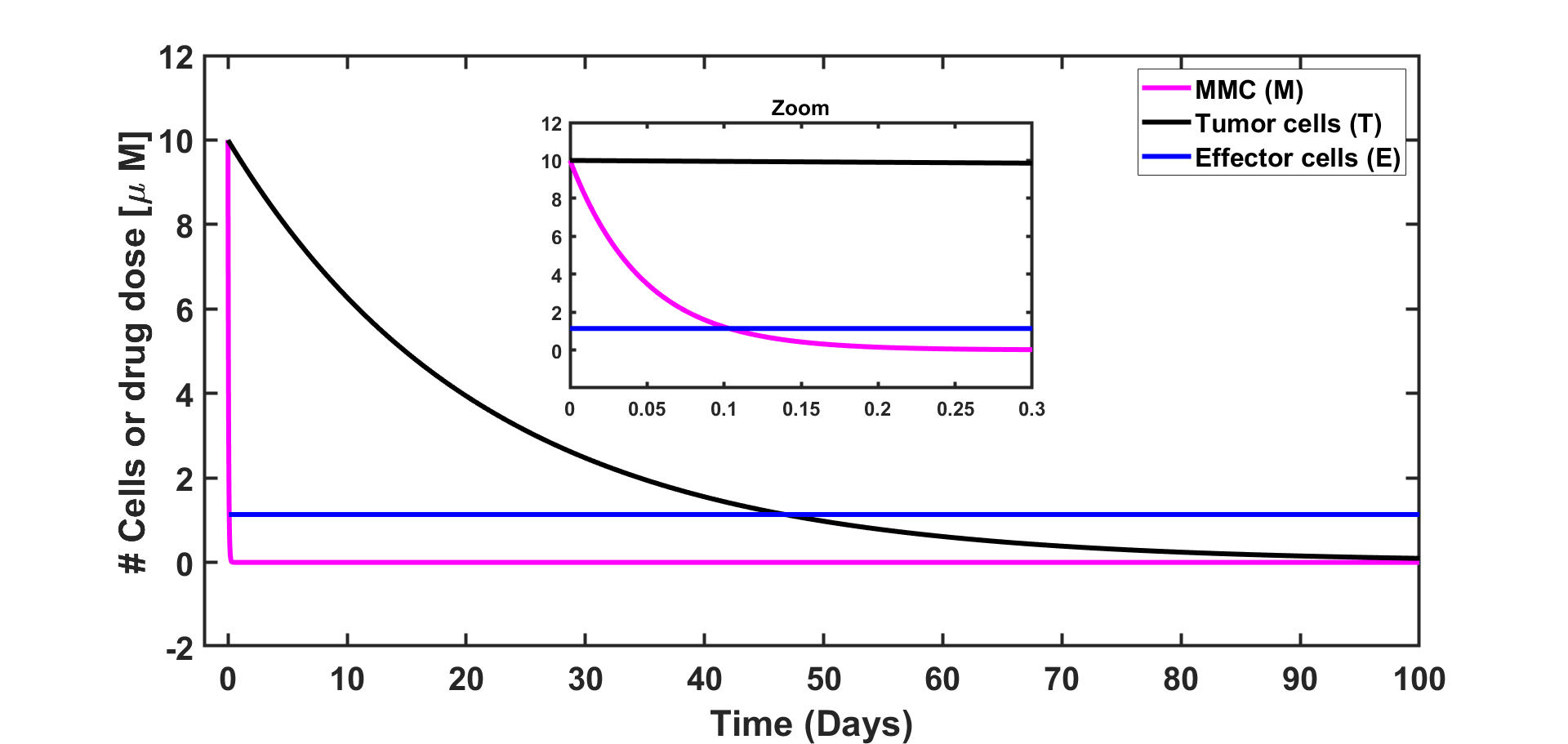

Supplement: Supplementary file 2 [file Image_1.png]

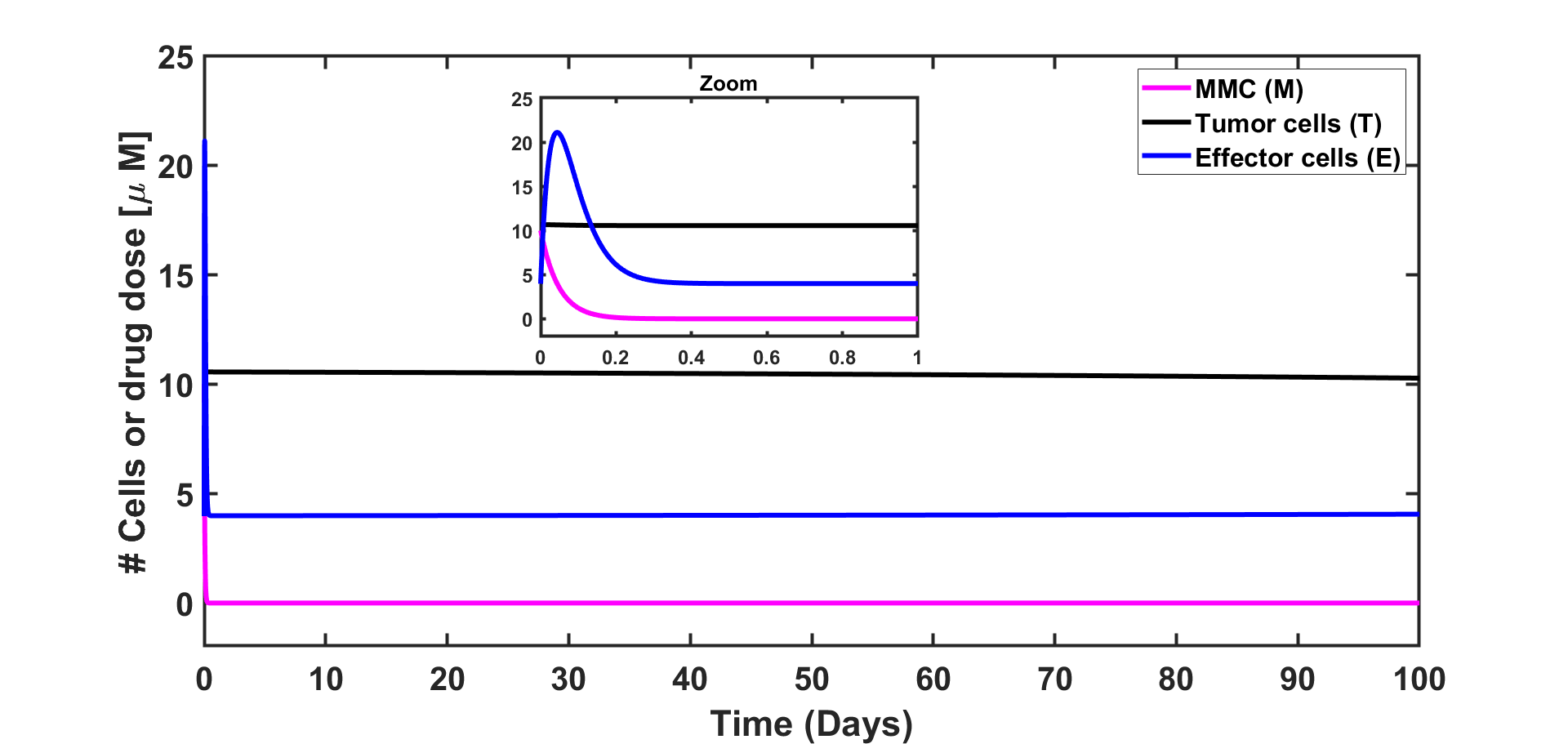

Supplement: Supplementary file 3 [file Image_2.png]

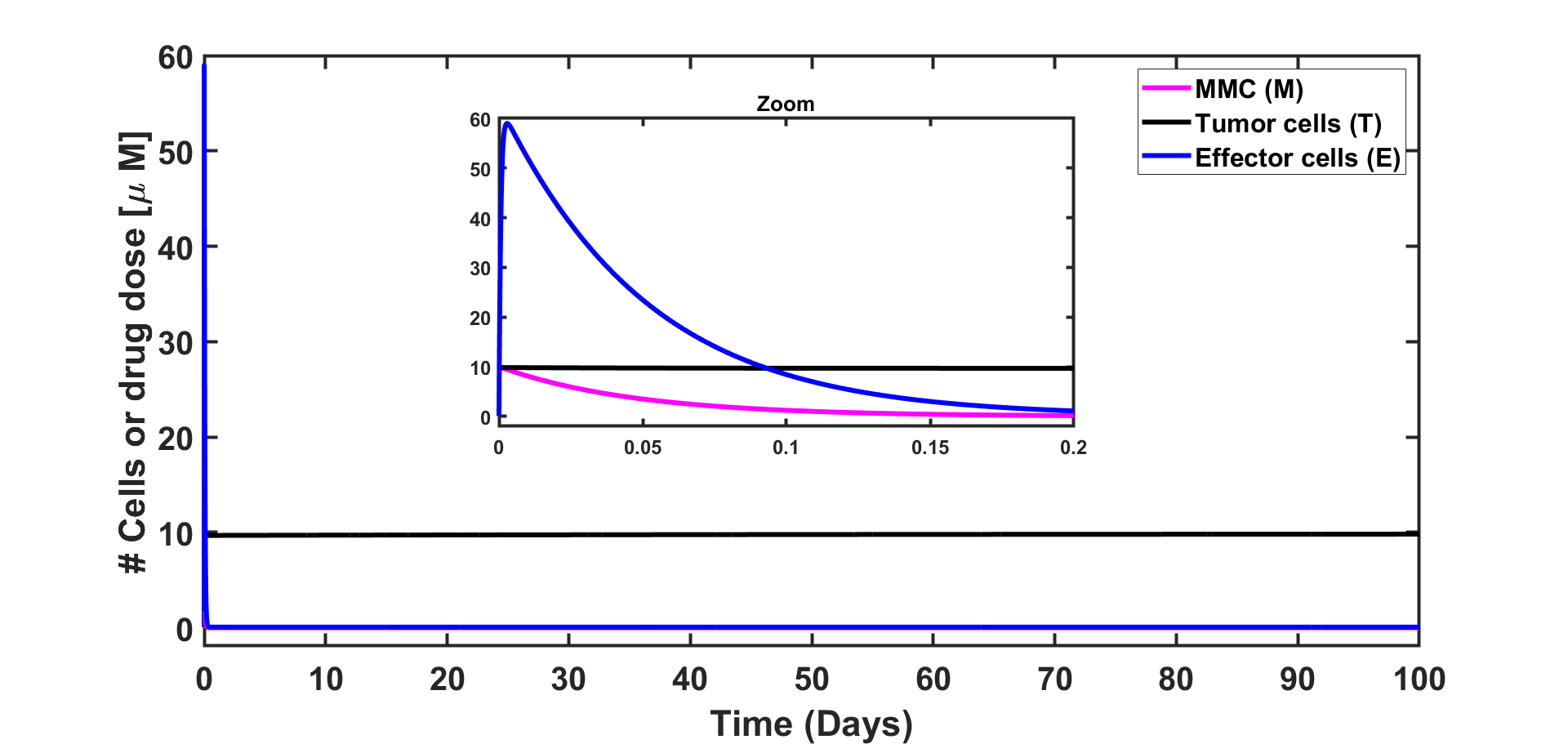

Supplement: Supplementary file 4 [file Image_3.png]

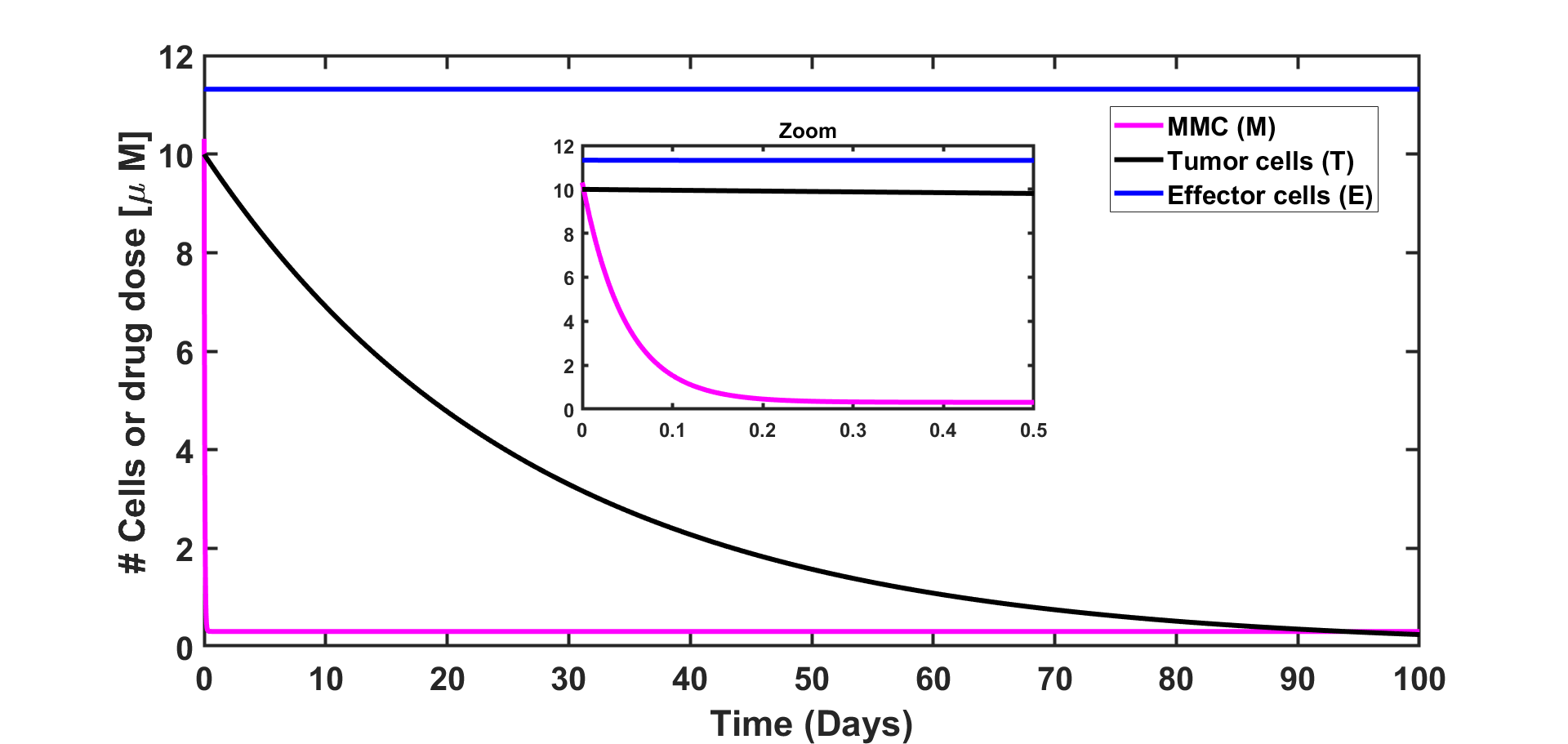

Supplement: Supplementary file 5 [file Image_4.png]

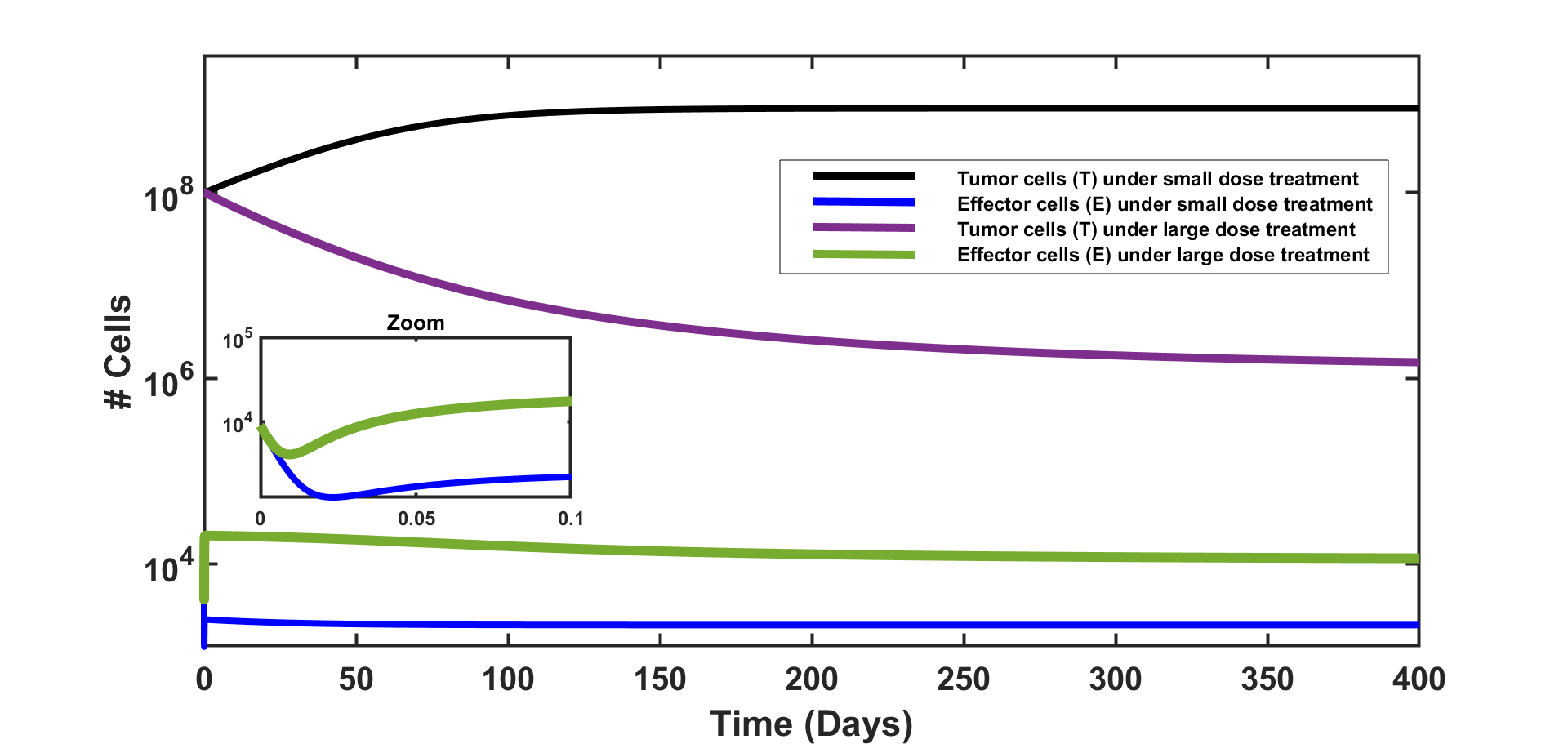

Supplement: Supplementary file 6 [file Image_5.png]

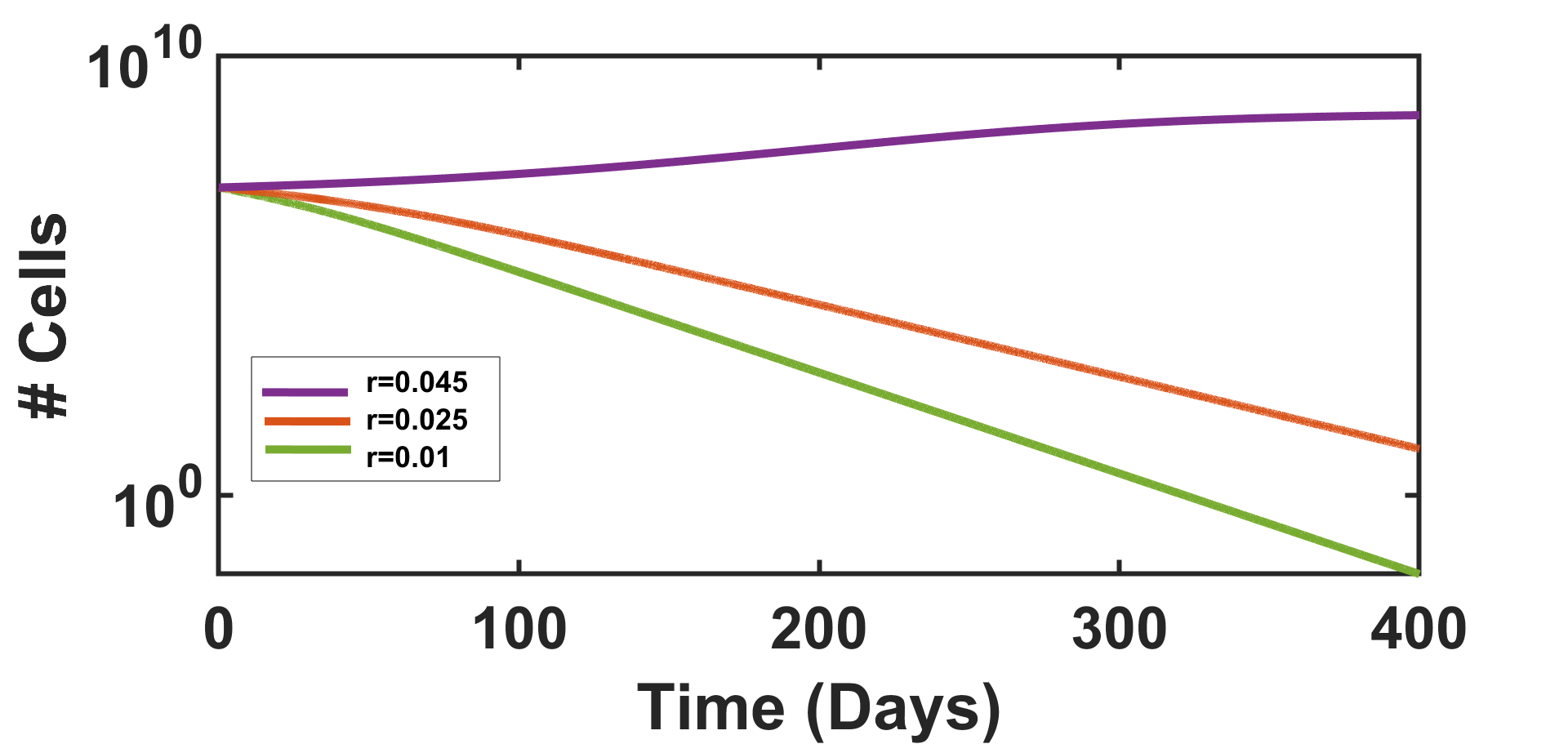

Supplement: Supplementary file 7 [file Image_6.png]

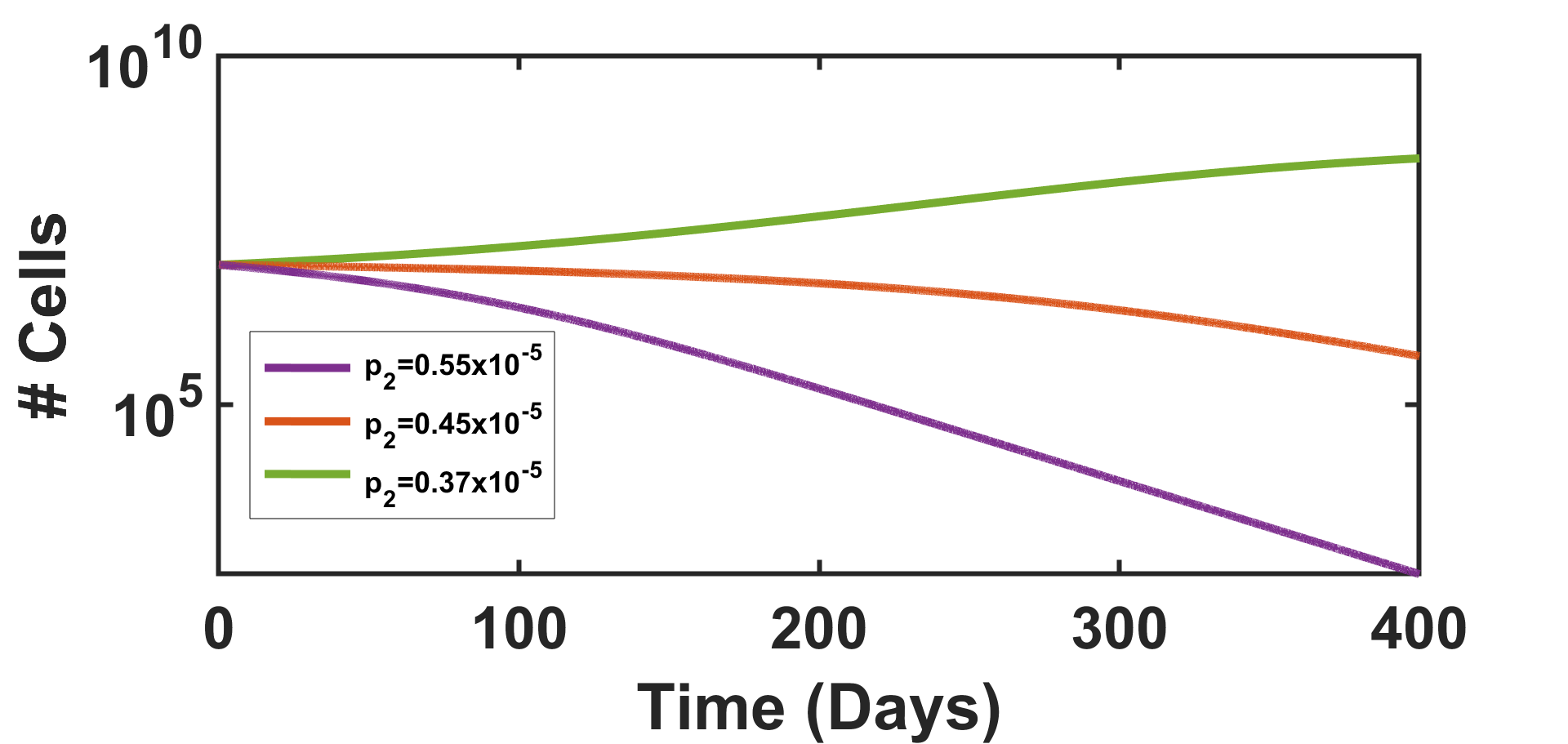

Supplement: Supplementary file 8 [file Image_7.png]

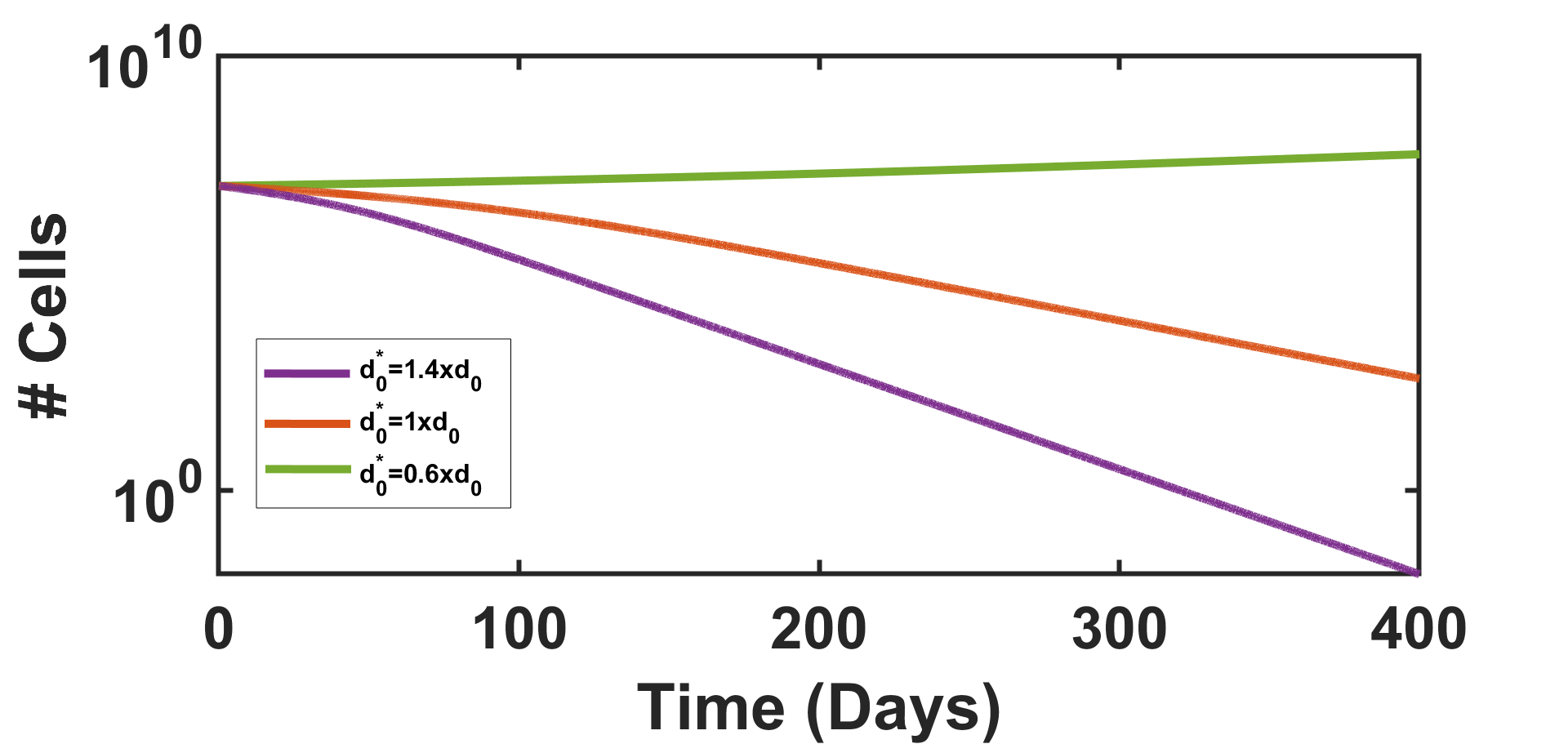

Supplement: Supplementary file 9 [file Image_8.png]

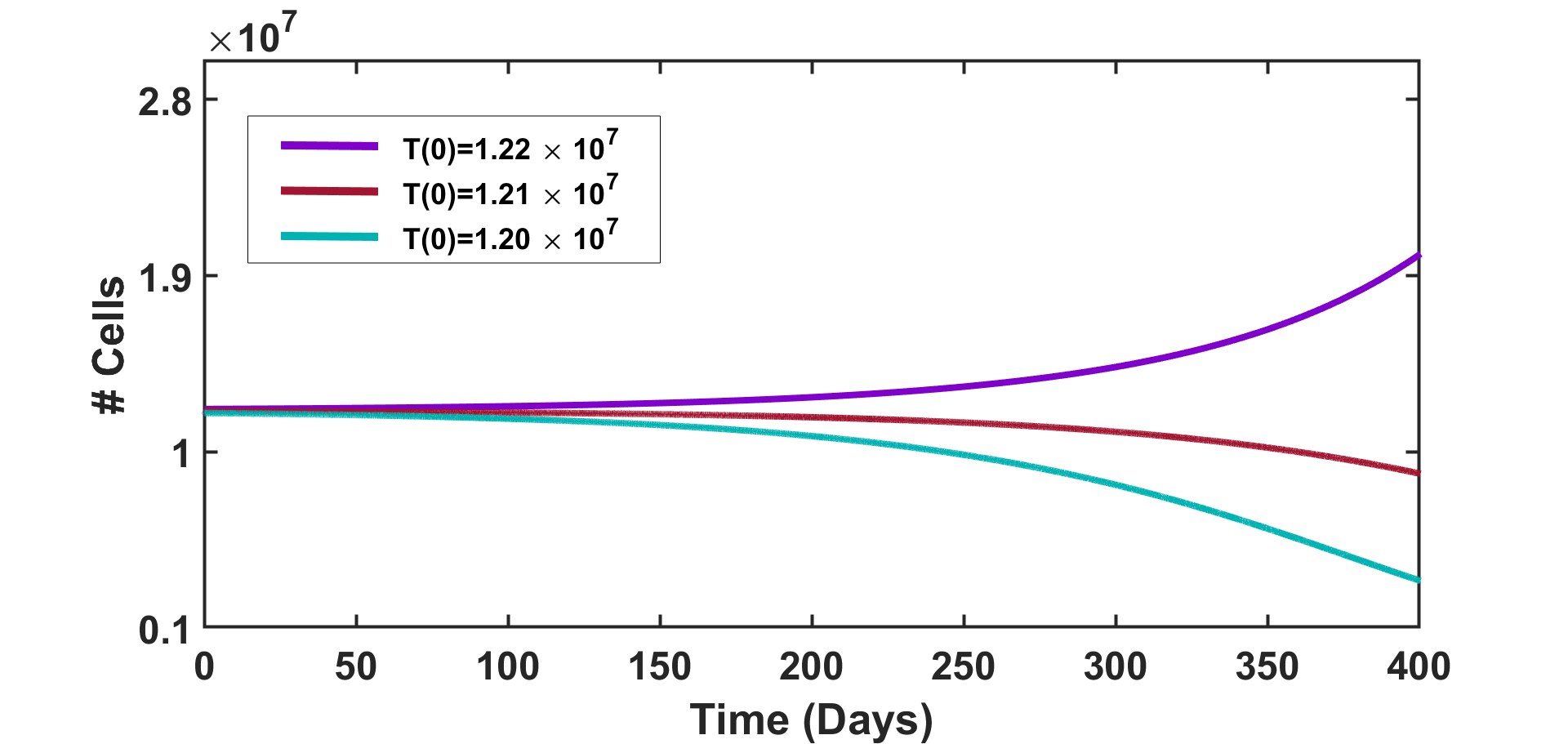

Supplement: Supplementary file 10 [file Image_9.png]

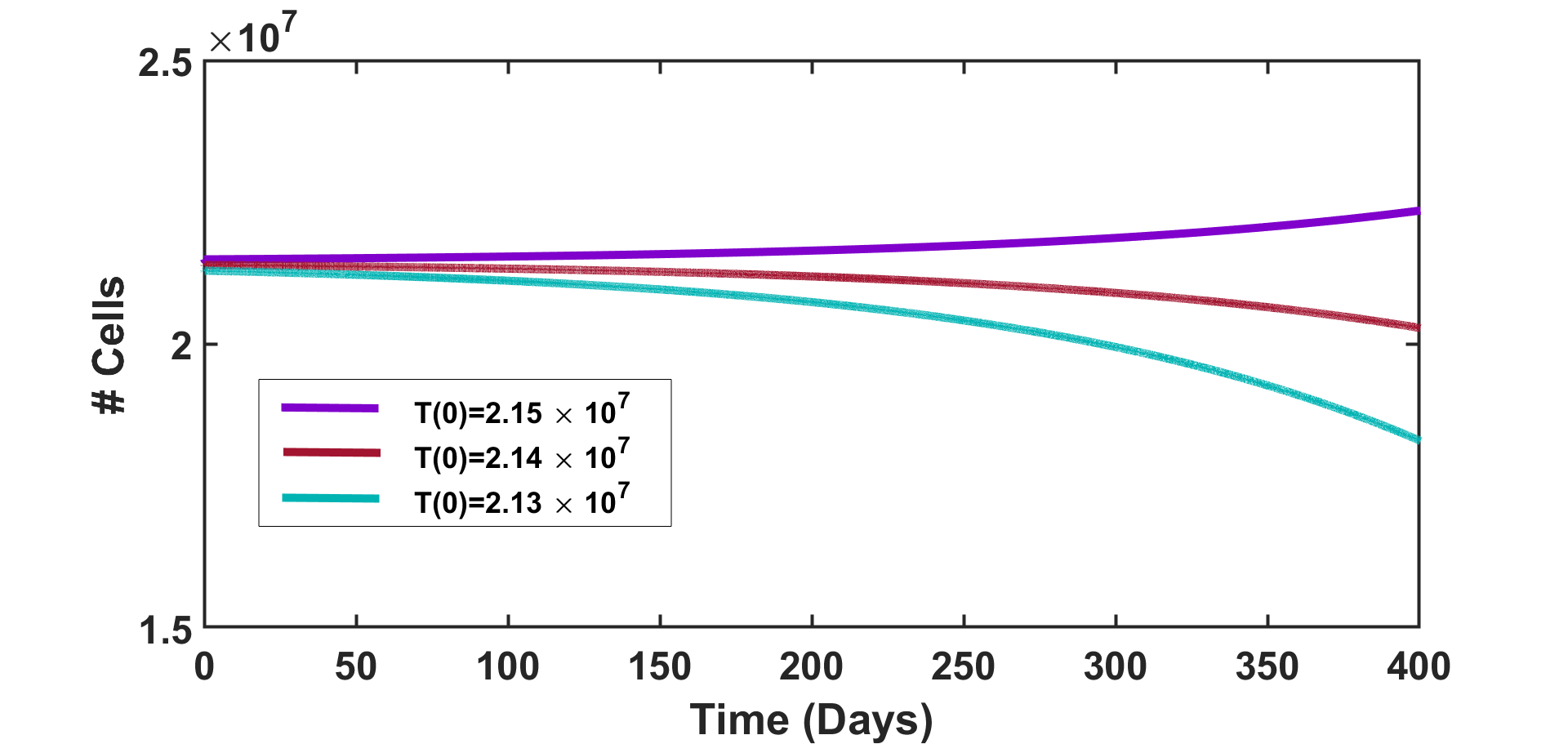

Supplement: Supplementary file 11 [file Image_10.png]

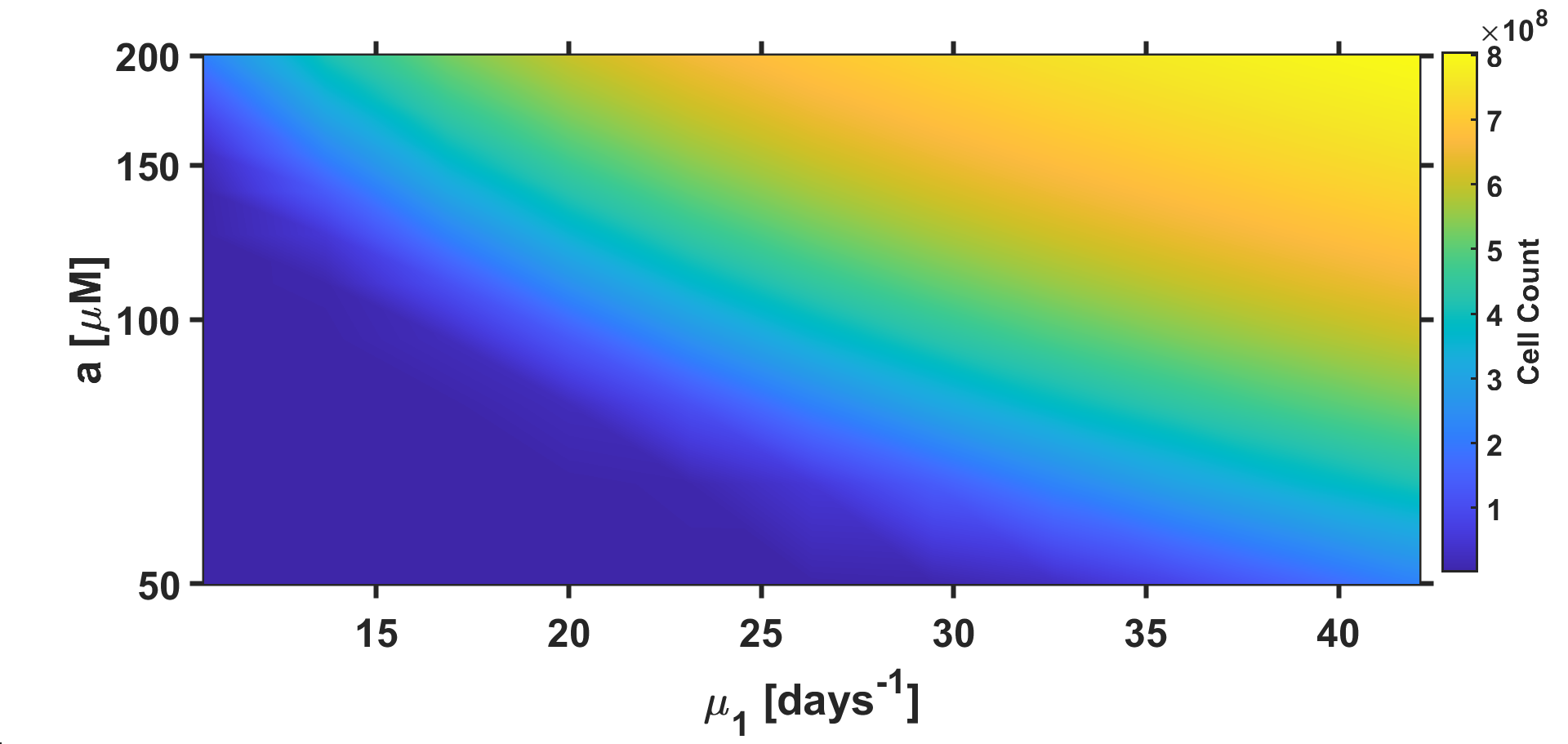

Supplement: Supplementary file 12 [file Image_11.png]
